# Genetic determinants of liking and intake of coffee and other bitter foods and beverages

**DOI:** 10.1038/s41598-021-03153-7

**Published:** 2021-12-13

**Authors:** Marilyn C. Cornelis, Rob M. van Dam

**Affiliations:** 1 Department of Preventive Medicine, Northwestern University Feinberg School of Medicine, 680 North Lake Shore Drive, Suite 1400, Chicago, IL 60611 USA; 2 Department of Exercise and Nutrition Sciences, Milken Institute School of Public Health, The George Washington University, Washington, DC USA; 3 Department of Epidemiology, Milken Institute School of Public Health, The George Washington University, Washington, DC USA; 4 Department of Nutrition, Harvard T.H. Chan School of Public Health, Boston, MA USA

**Keywords:** Genetics, Risk factors

## Abstract

Coffee is a widely consumed beverage that is naturally bitter and contains caffeine. Genome-wide association studies (GWAS) of coffee drinking have identified genetic variants involved in caffeine-related pathways but not in taste perception. The taste of coffee can be altered by addition of milk/sweetener, which has not been accounted for in GWAS. Using UK and US cohorts, we test the hypotheses that genetic variants related to taste are more strongly associated with consumption of black coffee than with consumption of coffee with milk or sweetener and that genetic variants related to caffeine pathways are not differentially associated with the type of coffee consumed independent of caffeine content. Contrary to our hypotheses, genetically inferred caffeine sensitivity was more strongly associated with coffee taste preferences than with genetically inferred bitter taste perception. These findings extended to tea and dark chocolate. Taste preferences and physiological caffeine effects intertwine in a way that is difficult to distinguish for individuals which may represent conditioned taste preferences.

## Introduction

Coffee and tea are among the most widely consumed beverages in the world^1^. The consumption of these plant-based beverages has been associated with a lower risk of chronic diseases such as type 2 diabetes, cardiovascular diseases, and several types of cancer^2–9^. Although plausible underlying biological mechanisms have been identified, more research is needed to establish the causal role of these beverages in human health^10^. Thus, understanding determinants of beverage choice and consumption level is important to inform research and public health strategies.

Genome-wide association studies (GWAS) of coffee and tea drinking behavior have identified genetic variants involved in the metabolism and physiological effects of caffeine as determinants of the amount of these beverages consumed (Supplementary Table S1)^11–20^. Furthermore, a variant near a gene encoding an olfactory receptor (*OR5M8*) has been associated with coffee intake. As coffee and tea have a bitter taste, it is also plausible that genetic variants related to bitter taste perception affect coffee and tea consumption. However, none of the loci identified in GWAS of coffee and tea intake overlap with *TAS2R* loci associated with taste perception of bitter compounds including propylthiouracil (PROP)/phenylthiocarbamide (PTC), caffeine, and quinine in GWAS (Supplementary Table S1)^22–25^. Caffeine seeking behavior might explain the persistent consumption of coffee and tea despite their bitter taste^21,26^. However, the taste of these beverages is easily manipulated by the addition of sweetener and milk; behaviors not previously accounted for in GWAS or the vast majority of epidemiological studies.

The current study uses genetic, dietary, and food preference (“liking”) data available from the UK Biobank (UKB) and two US cohorts, the Nurses’ Health Study (NHS) and Health Professionals Follow-up study (HPFS). We first test the hypothesis that published GWAS-confirmed variants related to taste are more strongly associated with black coffee consumption than with the consumption of total coffee or coffee with added sweetener or milk because their effects would not be masked by coffee taste manipulation. We focus on coffee but extend this hypothesis to tea since we expect similar but weaker associations of taste-related genetic variants with tea consumption, because tea is reportedly less bitter than coffee^27,28^. As a negative control, we also examine published GWAS-confirmed loci involved in the metabolism and physiology of caffeine which we would not expect to be differentially associated with type of coffee and tea consumed independent of caffeine content. Second, we examine whether there are shared genetic determinants of coffee and tea traits with other bitter tasting foods; specifically, beer and dark chocolate. Finally, we perform GWAS of liking or consumption of specific types of coffee and tea (e.g., with sugar versus without sugar) that may yield genetic variants not reported previously by GWAS of total coffee and tea consumption.

## Methods

### UK biobank

In 2006–2010, the UKB recruited over 502,633 participants aged 37–73 years at 22 centers across England, Wales, and Scotland^29^. Participants provided informed written consent, completed touchscreen questionnaires on sociodemographic factors, lifestyle, and medical history followed by an interviewer-administered questionnaire, physical assessment, and biospecimen collection^30^. Subsets of the cohort have returned for follow-up assessments and have completed additional on-line questionnaires. The latter are the primary source of data for the current analysis. This study was covered by the generic ethical approval for UKB studies from the National Research Ethics Service Committee North West–Haydock (approval letter dated 17th June 2011, Ref 11/NW/0382), and all study procedures were performed in accordance with the World Medical Association Declaration of Helsinki ethical principles for medical research.

#### Coffee and tea consumption

In 2009–2012, a subset of 122,292 participants who completed the baseline assessment center visit also completed at least two of five on-line 24 h dietary recalls^31,32^. Detailed collection methods for coffee and tea intake and methods for estimating added milk and sweetener are provided in Supplementary Methods S2. Briefly, participants reporting consumption of coffee were probed for details concerning quantity (0.5–6 + cups/day), brew type (instant, filtered, espresso, cappuccino, latte, and other), and whether the coffee was decaffeinated (yes, no, varied) and sweetened (half, 1, 2, 3 + teaspoons or “varied” for sugar and artificial sweetener). Categorical measures of coffee quantity were converted to cups/day by using the midpoint of each category; those reporting 6 + cups/day were assigned an intake of 6 cups/day. Those reporting consumption of instant, filtered, espresso or other coffee were additionally asked if milk was added (yes, no, varied). Participants reporting consumption of tea were probed for details concerning quantity (1–6 + cups/day), brew type (green, black, rooibos, herbal and other), and whether it was decaffeinated (black tea only) and sweetened. Those reporting consumption of black or rooibos tea were additionally asked if milk was added. We also considered responses to the following bitter tasting food items: "How many pints of beer, lager or cider?", "How many plain/dark chocolate bars (~ 50 g) did you have?", "How many servings of sprouts did you have?", and "How many servings of cabbage/greens/kale did you have?" which aligned with food preference items of interest (see below). Data collected via dietary recalls also allowed derivation of the energy content (KJ) and macronutrient composition of the diet. *Unsweetened coffee* consumers were defined as participants who never reported the addition of sweetener to coffee and never reported consumption of cappuccinos or lattes. *No-milk coffee* consumers were defined as participants who never reported the addition of milk to coffee and never reported consumption of cappuccinos or lattes. *Black coffee* consumers were those previously defined as both unsweetened and no-milk coffee consumers. *Sweetened coffee* consumers were defined as participants who always reported the addition of sweetener to coffee or who only consumed cappuccinos or lattes. *Milk coffee* consumers were defined as participants who always reported the addition of milk to coffee or who only consumed cappuccinos or lattes. Consumption data for coffee drinkers not defined as milk coffee consumers were set missing for genetic analysis but set to 0 for trait correlation analysis; the same approach was applied to other coffee consumption traits. Similar criteria were applied when defining different tea consumers while considering the relatively less detailed data collected for this beverage. Herein, *tea-prepared-black* refers to tea prepared without sweetener and milk to avoid confusion with tea type (i.e., black, green). The comprehensive list of coffee and tea traits account, in part, for cultural differences in how these beverages are prepared between UK and US cohorts.

#### Coffee and tea preferences

In 2019, UKB participants with valid emails were invited to complete a food preferences questionnaire (see Supplementary Methods S2 for details). The questionnaire included 140 items which comprise food items that reflect both sensory preferences and foodstuff preferences. Liking was measured using a 9-point hedonic scale which has good statistical properties, good discrimination between points, and linearity between each point on the scale^33^. Questionnaire items were randomized on a participant basis to reduce any bias that may occur due to tiredness. Participants were asked to rate how much he/she like each presented item on a scale from 1 (extremely dislike) to 9 (extremely like). Alternatively, they were given the option to select “Never tried” or “Prefer not to answer”. The current study focused on the following questionnaire items: liking for coffee with sugar, liking for coffee without sugar, liking for tea with sugar, and liking for tea without sugar. We also considered other bitter- and sweet-related items: liking for bitter foods, liking for dark chocolate, liking for Brussel sprouts, liking for cabbage, liking for bitter/ale and liking for sweet foods. In preliminary analysis, (1) liking for bitter vegetables was only weakly correlated with intake of bitter vegetables (r < 0.16) and liking for bitter foods (r < 0.10) and (2) liking for or intake of bitter vegetables were only weakly associated with coffee and tea traits (r < 0.15). Therefore, we did not pursue genetic analysis of bitter vegetable traits as results would unlikely be relevant for coffee and tea traits. After excluding 31 participants with liking score ranges of less than 4 across all 140 food items (an indicator of scale bias), up to 181,974 participants had data on food items of interest for the current analysis.

#### Genetic data

All UKB participants were genotyped using genome-wide arrays as detailed previously^34,35^. QC and imputation to the HRC v1.1 and UK10K reference panels was performed by the Wellcome Trust Centre for Human Genetics^35^. We excluded sample outliers based on heterozygosity and missingness, participants with sex discrepancies between the self-reported and X-chromosome heterozygosity, and those potentially related to other participants, based on estimated kinship coefficients for all pairs of samples. To avoid bias due to population stratification, genetic analysis performed in the current study were limited to unrelated individuals who self-report as “British” and who have very similar ancestral backgrounds based on results of principal component (PC) analysis^35^.

Of this UKB genetic sample, up to 126,599 individuals completed coffee- or tea-related liking scales and up to 86,006 participants had detailed coffee or tea consumption data based on 24-h recalls. Up to 61,955 participants had both liking and dietary intake data.

#### Other covariates

Self-reported smoking status, physical activity, Townsend deprivation index, education, income, employment status as well as technician-measured body weight and height were collected during the UKB baseline assessment as described in detail previously^29,36^.

### US cohorts

In 1986, the HPFS enrolled 51,529 U.S. male health professionals aged 40–75 years^37^. In 1976, the NHS enrolled 121,700 U.S. female registered nurses aged 30–55 years^38^. Participants completed a mailed questionnaire on medical history and lifestyle characteristics every 2 years and a validated semi-quantitative food frequency questionnaire (FFQ) every 2–4 years^39^. All participants provided informed consent and study protocols were approved by the institutional review boards of Brigham and Women's Hospital and Harvard School of Public Health.

#### Coffee and tea consumption

We considered diet data collected by the FFQ administered closest to and before the 2018 supplementary questionnaire described below. For NHS this was the 2010 FFQ and for HPFS the 2014 FFQ. For each FFQ item, participants were asked how often, on average, they had consumed a specified amount of each beverage or food over the past year. The participants could choose from nine frequency categories (never, 1–3 per month, 1 per week, 2–4 per week, 5–6 per week, 1 per day, 2–3 per day, 4–5 per day, and 6 or more per day). Categorical measures of intake were converted to servings or cups/day by using the midpoint of each category; those reporting 6 or more servings or cups/day were assigned a daily intake of 6. The current analysis focused on coffee (regular or decaf) and tea (regular or decaf, not herbal) but also considered beer and dark chocolate as done for UKB. Total dietary energy intake and macronutrient composition of the diet were also derived from FFQs.

#### Coffee and tea drinking behaviors

In 2018, more detailed questions regarding coffee and tea drinking behavior were included on a supplementary questionnaire mailed to NHS and HPFS participants previously selected for GWAS whom had not completed a supplementary questionnaire in 2010^40^. Participants were asked “How do you usually drink your coffee or tea?” and for each beverage he/she could mark all response items as appropriate: “I do not drink this beverage”, “Black (nothing added)”, “Milk or cream”, “Non-dairy creamer/whitener”, “Sweetener (e.g., sugar, honey, syrup)”, “Non-caloric sweetener (e.g., Splenda, Equal, stevia)”. Participants were also asked “Do you avoid or drink less coffee because it tastes bitter?” (yes or no), “Do you avoid or drink less tea because it tastes bitter?” (yes or no). After one mailing, 5173 NHS (80% response) and 2940 HPFS (70%) returned the questionnaire.

#### Genetic data

Genetic data contributing to the current study were obtained from independent GWAS case–control studies nested within the cohorts, initially designed for outcomes of type 2 diabetes, coronary heart disease, gout, kidney stone, open-angle glaucoma, venous thromboembolism, prostate cancer (HPFS only), pancreatic cancer, colon cancer, mammographic density (NHS only), endometrial cancer (NHS only), ovarian cancer (NHS only) and breast cancer (NHS only). Studies were genotyped on Affymatrix, Illumina, Omni, OncoArray or HumanCoreExome platforms^41^. To allow for maximum efficiency and power, we pooled HPFS and NHS samples genotyped on the same platforms and for each of the resulting datasets we imputed SNPs based on the 1000 Genomes (version 1.1 2016) cosmopolitan reference panel. Detailed methods and quality assurance pertaining to these genetic datasets have been reported elsewhere^41^. Any samples that had substantial genetic similarity to non-European reference samples were excluded from genetic analysis.

Of the participants with high-quality genetic data, 4295 NHS and 2447 HPFS participants had survey data on coffee or tea behaviors. Because these data were used in conjunction with FFQ data to derive a quantity of specific type of coffee consumed that aligned with that derived for UKB (see above for definitions), we excluded 286 HPFS and 175 NHS who did not complete an FFQ. We also excluded coffee data from 130 HPFS and 246 NHS who changed coffee drinking status (yes/no) between the FFQ and survey. The same approach was taken for tea data resulting in the exclusion of 497 HPFS and 836 NHS participants. In total, up to 2123 HPFS and 4064 NHS participants with genetic and coffee or tea data were included for the current analysis.

#### Other covariates

Smoking status, physical activity, height, and body weight of participants were self-reported and obtained from questionnaires administrated to the entire NHS (2014) and HPFS (2016) cohorts preceding the 2018 supplementary questionnaire.

### Candidate SNP selection

We first selected GWAS-confirmed SNPs for taste perception of PROP/PTC, caffeine, and quinine^22–25^ (Supplementary Table S1); herein referred to as “Taste-loci”. Genetically inferred PROP-taster status was defined using rs1726866, rs713598 and rs10246939: AVI/AVI, PAV/PAV were coded as 0 (non-taster) and 2 (super taster), respectively, while all other haplotypes were coded 1 (medium taster). We next selected GWAS confirmed SNPs for liking or consumption of coffee and tea^11–20^, herein referred to as “Behavior-loci”. Because *ADORA2A* variants were among this list we additionally included *ADORA2A* rs5751876, an often-cited variant associated with caffeine consumption and caffeine-induced anxiety and wakefulness^42–45^. We also included a *CYP2A6* variant associated with paraxanthine/caffeine plasma levels in GWAS and also with coffee consumption in UKB^46^.

### Statistical analysis

All statistical analyses were performed using the SAS statistical package (version 9.1 for UNIX; SAS Institute, Cary, NC) unless indicated otherwise. Potential bias in use of the 9-point liking scale (unrelated to the content of the items) was evaluated by comparing mean scores of all 140 food items by age (at or above/below the median of 67 years), sex, smoking and BMI (at or above/below 25 kg/m^2^) using ANOVA. Significantly higher mean food liking scores were reported by participants who were younger, male, and had lower BMI compared to their respective counterparts (P < 0.0001). Because mean score differences were small, ranging from 0.19 (sex) to 0.03 (age), we only adjusted for these factors in our primary analyses as opposed to performing stratified analyses. The distributions of all coffee- and tea-related traits of interest were highly skewed and thus non-parametric tests were applied. Bivariate Spearman correlations were used to evaluate correlations among traits.

For UKB, multivariable-adjusted generalized linear modelling (GLM) was used to examine the association between each SNP (independent variable) and each continuous coffee/tea trait (dependent variable), adjusting for age, sex, smoking status, genotyping array and the top 20 PCs. Additional adjustments for BMI, physical activity, education level, Townsend index of socio-economic status, employment status, self-reported diabetes and heart disease and intake of total energy, alcohol, and other macronutrients (expressed as a proportion of energy) did not substantially change the results and thus we present results for the more basic model only. For NHS and HPFS, GLM was also used to examine the association between each SNP and each continuous coffee/tea trait, adjusting for age, smoking status, genotyping array and GWAS-specific case–control status. Results for NHS and HPFS were meta-analyzed with fixed effects using METAL^47^. We applied the same statistical models defined above to the analysis of coffee (tea) avoiders due to bitter taste (yes vs no) using a logistic regression analysis. Given the highly correlated coffee and tea traits as well as the confirmatory and hypothesis testing nature of the current analysis, statistical significance was defined as P < 0.002 (0.05/23 SNPs) for both UKB and US (NHS/HPFS) cohorts; correcting only for the number of independent SNPs tested. Differences between beta-coefficients (i.e. β_SNP-total coffee_ vs β_SNP-black coffee_ or β_SNP-black coffee_ vs β_SNP-sweetened coffee_) in UKB were declared significantly different when their corresponding 95% confidence intervals did not overlap; an approach considered highly conservative^48^. Nevertheless, emphasis is placed on results consistent across UK and US cohorts.

We performed GWAS of continuous coffee and tea traits in UKB (excluding total coffee/tea). The rank-based inverse (blom) normal transformation was applied to each trait prior to GWAS and we excluded SNPs with MAF < 0.05 and INFO scores < 0.4. We performed genome-wide linear regressions using PLINK2 assuming an additive genetic model and adjusting for age, sex, smoking status and top 20 PCs. FUMA was used for displaying, pruning and annotating UKB summary-level results^49^. Genome-wide significant (P < 5 × 10^–8^) SNP-trait associations were followed up in NHS and HPFS when possible and using statistical models as described above for candidate SNP-analysis.

## Results

### Participant characteristics

Table 1 presents the characteristics of UKB, NHS and HPFS participants by coffee drinking status; 82, 86, and 85% of the cohort participants, respectively, were coffee drinkers (consuming more than 0 cups/day). Across cohorts, coffee drinkers were 1–2 years older and more likely to be male and consume more alcohol and beer than non-coffee drinkers. In the UKB and NHS, coffee drinkers were also more likely to be current smokers. HPFS non-coffee drinkers were more likely to be current smokers. Supplementary Table S2 presents corresponding data by tea drinking status; 87, 82 and 65% of UKB, NHS, and HPFS participants, respectively, were tea drinkers (consuming more than 0 cups/day). Across cohorts, tea drinkers were more likely to be female and non-smokers and to consume less alcohol and beer. In the UKB, tea drinkers were also less likely to be overweight.

**Table 1:** Characteristics by coffee drinking status.

Variable	UK Biobank		NHS		HPFS	
	Non-drinkers n = 15,489	Drinkers n = 70,517	Non-drinkers n = 517	Drinkers n = 3357	Non-drinkers n = 305	Drinkers n = 1726
Age at diet collection, years	56.6 ± 7.9	58.5 ± 7.7	72.9 ± 6.0	74.0 ± 5.9	76.8 ± 6.6	77.3 ± 6.5
Age at liking/preference collection, years	64.8 ± 7.7	66.8 ± 7.5	80.5 ± 5.9	81.6 ± 5.9	80.8 ± 6.6	81.3 ± 6.5
Female, n (%)	9316 (60)	38,550 (55)	517 (100)	3357 (100)	0 (0)	0 (0)
Current smoker, n (%)	859 (6)	4870 (7)	12 (2)	108 (3)	125 (41)	20 (1)
BMI, kg/m^2^	26.7 ± 4.8	26.7 ± 4.5	26.9 ± 5.7	26.5 ± 5.1	26.0 ± 4.4	26.1 ± 3.8
BMI ≥ 30 kg/m^2^, n (%)	3101 (20)	13,581 (19)	124 (24)	719 (21)	41 (13)	243 (14)
Alcohol intake, g/day	13.9 ± 19.2	16.9 ± 18.5	3.1 ± 7.3	7.7 ± 11.7	7.4 ± 13.3	15.5 ± 16.3
Energy intake, Kcal/day	2001 ± 445	2053 ± 430	1687 ± 563	1639 ± 529	1949 ± 557	2051 ± 579
Total coffee, cups/day	–	2.1 ± 1.3	–	1.9 ± 1.3	–	2.0 ± 1.4
Coffee preference, n (%)						
Black	–	9034 (13)	–	1164 (35)	–	824 (48)
Unsweetened	–	37,615 (53)	–	2495 (74)	–	1241 (72)
No-milk	–	11,102 (16)	–	1359 (40)	–	974 (56)
Sweetened	–	22,265 (32)	–	891 (27)	–	495 (29)
Milk	–	50,360 (71)	–	2027 (60)	–	762 (44)
Total tea, cups/day	3.8 ± 1.8	2.4 ± 1.7	1.4 ± 1.6	0.7 ± 1.0	0.6 ± 1.1	0.5 ± 0.8
Avoid/drink less coffee because bitter^a^, n %	–	–	180 (42)	286 (9)	88 (35)	131 (8)
Dark chocolate, servings/day	0.04 ± 0.15	0.04 ± 0.15	0.12 ± 0.27	0.08 ± 0.21	0.13 ± 0.26	0.14 ± 0.29
Beer, servings/day	0.30 ± 0.71	0.32 ± 0.68	0.02 ± 0.14	0.04 ± 0.20	0.18 ± 0.62	0.26 ± 0.52

Shown are mean ± SD for continuous variables or n (%) for categorical variables. Non-drinkers are defined as consuming no coffee. Drinkers are defined as consuming any coffee (> 0 cups/day). Coffee preference type was derived from diet-recalls in UKB and a combination of food frequency questionnaires and supplemental beverage surveys in NHS/HPFS (see “Methods” for details).

^a^Missing data for 145 NHS and 64 HPFS participants. This information was not collected in UKB.

**Figure 1:**
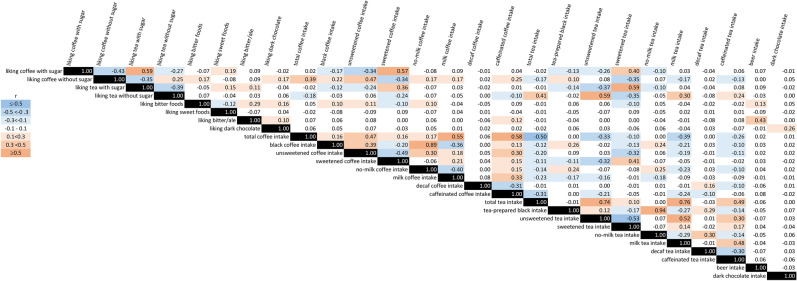
UKB trait spearman correlations.

The consumption of coffee and tea prepared black was more common in the US cohorts than the UKB, because US participants were less likely to add milk to these beverages than UKB participants (Table 1, Supplementary Table S2). Correlations among coffee, tea and other diet behavior traits for UKB are presented in Fig. 1 (details in Supplementary Table S3). Corresponding correlations for NHS and HPFS are presented in Supplementary Tables S4 and S5, respectively. In UKB, liking coffee and tea traits were generally moderately (r = 0.5–0.7) correlated with the respective intake trait (e.g., liking coffee with sugar correlated with sweetened coffee intake). Total coffee and total tea intake were each more strongly associated with liking of coffee and tea without sugar (r > 0.3), than with liking coffee and tea with sugar (|r|< 0.01). In the UKB, liking bitter foods was more strongly correlated with liking coffee without sugar (r = 0.17) and intakes of black (r = 0.10) or unsweetened (r = 0.11) coffee than with liking coffee with sugar (r = − 0.07) and intakes of sweetened (r = − 0.10) or milk (r = − 0.04) coffee. Liking bitter foods was also positively correlated with liking dark chocolate (r = 0.16) and negatively correlated with sweetened tea intake (r = − 0.10).

Candidate “Taste” and “Behavior” loci selected for the current analysis are presented in Supplementary Table S1 and, with the exceptions of rs5751876 (*ADORA2A)*, are previously reported GWAS-confirmed loci for indicated traits. Following, we present associations between our novel consumption/liking traits and i) GWAS-confirmed loci for bitter *taste* perception and ii) GWAS-confirmed loci for coffee/tea consumption *behavior*. We annotate statistically significant (P < 0.002) associations in Tables and note nominally significant (0.002 < P < 0.05) associations below. We then present results from GWAS of our novel consumption/liking traits in UKB applying the traditional GW-significance threshold (P < 5 × 10^–8^) along with replication in NHS/HPFS.

### GWAS-confirmed loci for bitter taste perception

Associations between genetic variants related to taste perception and the liking and intake of different types of coffee, tea, and other bitter foods in UKB are shown in Table 2 (details in Supplementary Table S6). Related results for the US cohorts, NHS and HPFS, were pooled and shown in Supplementary Table S7. The quinine-taste sensitive variant near *TAS2R19* (rs10772420 A) was significantly (P < 0.002) inversely associated with liking coffee with sugar (β = − 0.04) in UKB. Similarly, the variant was nominally (0.002 < P < 0.05) associated with less liking of tea with sugar (β = − 0.03) and greater liking of tea without sugar (β = 0.03). In UKB, the variant was nominally associated with lower coffee intake (β = − 0.02), regardless of the type of coffee. In the US cohorts, the variant was nominally significantly associated with higher intake of black (β = 0.12), unsweetened (β = 0.07), and no-milk (β = 0.10) coffee, but not substantially with total or other types of coffee. Although the *TAS2R19* variant was significantly associated with less liking of dark chocolate (β = − 0.03) in UKB, it was not associated with intake of tea, beer, or dark chocolate in any of the cohorts.

**Table 2:**
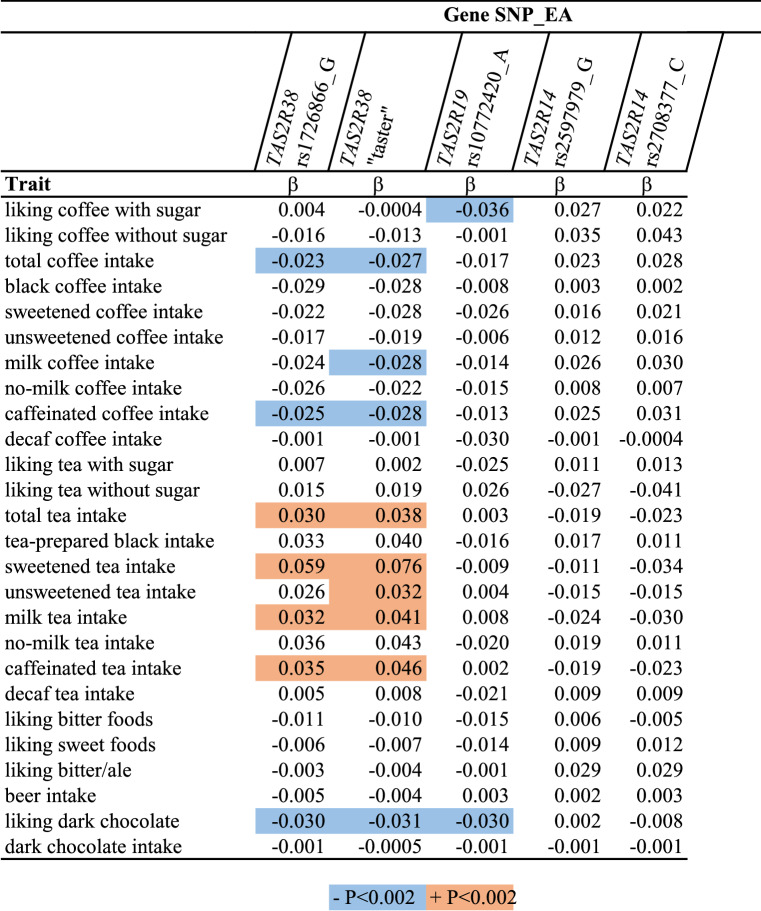
Bitter taste perception loci associations with coffee and other traits in UKB.

Shown are β-coefficients from linear regressions between SNPs (independent variable) and traits (dependent variable).

*EA* effect allele.

PROP-taste sensitive *TAS2R38* variants were also inversely associated with liking dark chocolate (β = − 0.03, P < 0.002), but not associated with liking coffee, tea, or bitter foods. However, the same variants were significantly associated with lower coffee intake (β = − 0.02), and higher tea intake (β = 0.03). The effect estimates were greatest for sweetened tea (β = 0.06) but not significantly different from other tea types. Results in the US cohorts were directionally consistent for coffee and tea intake but were not significant.

The caffeine-taste sensitive *TAS2R14* alleles were nominally associated with higher liking for coffee and intake of caffeinated coffee, regardless its preparation type. In the US cohorts this variant was nominally associated with higher intake of dark chocolate (β = 0.01, P < 0.05) but not with intake of coffee or tea.

### GWAS-confirmed loci for coffee/tea consumption behavior

Variants near *TMEM18, GCKR, POR, ADORA2A (*rs2330783*), CYP1A2 (*rs2472297, rs762551*), AHR, CYP2A6, SEC16B, OR5M7P, ENSA*, and *MLXIPL* were significantly associated with total coffee intake (P < 0.002, Table 3, details in Supplementary Table S8); consistent with previous GWAS (Supplementary Table S1). In addition, variants near *ABCG2*, *MC4R* and *AKAP6* were nominally associated with total coffee intake (0.002 < P < 0.05). Variants near *AHR* (rs4410790 C)*, CYP1A2* (rs2472297 C), and *OR5M7P* (rs597045 A) were more strongly associated with higher intakes of caffeinated than decaffeinated coffee intake. Variants near *AHR* (rs4410790 C)*, CYP1A2* (rs2472297 C), *ABCG2* (rs1481012 A)*, ADORA2A* (rs2330783 G)*, CYP2A6* (rs56113850 C)*, MC4R* (rs66723169 A)*, SEC16B* (rs574367 T)*, POR* (rs17685 A) and *TMEM18* (rs10865548 G) were exclusively or more strongly associated with *liking* coffee without sugar (P < 0.002) than liking coffee with sugar, but their association with higher coffee *intake* did not vary substantially by preparation type. Variants near *ALDH2, EFCAB, FIBIN, NRCAM, PDSS2*, and *BDNF* were not associated with coffee consumption in the current UKB sample, regardless of how coffee was usually prepared.

**Table 3:**
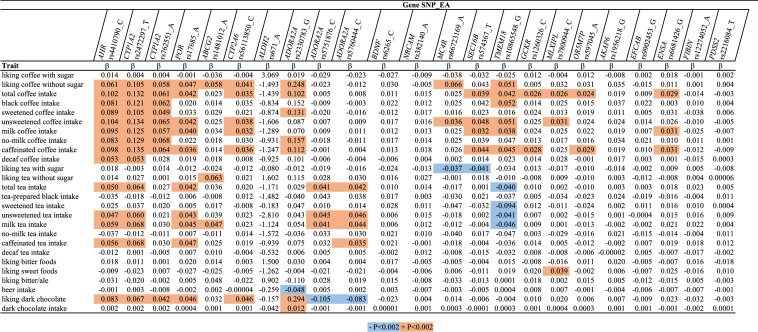
Coffee consumption behavior loci associations with coffee and other traits in UKB.

Shown are β-coefficients from linear regressions between SNPs (independent variable) and traits (dependent variable).

*EA* effect allele.

Variants near *AHR* (rs4410790 C) and *CYP1A2* (rs2472297 C) were also significantly associated with higher total tea intake (P < 0.002). This association was stronger for tea with milk than for tea-prepared black, and stronger for caffeinated than for decaffeinated tea. *TMEM18* (rs10865548 G) was significantly associated with lower tea intake and *ADORA2A* (rs5751876 C) was significantly associated with higher tea intake regardless of how it was prepared. *ABCG2* (rs1481012 A) was significantly associated with liking tea without (β = 0.06) but not with sugar, and was associated with higher tea intake regardless the type. *MC4R* (rs66723169 A) and *SEC16B* (rs574367 T) were significantly associated with less liking of tea with sugar (β = − 0.04), but neither was significantly associated with tea intake.

Variants near *ADORA2A* (rs2330783 G)*, AHR* (rs4410790 C)*, CYP1A2* (rs2472297 C)*, CYP2A6* (rs56113850 C)*,* and *POR* (rs17685 A) associated with higher coffee intake in published GWAS were also significantly (P < 0.002) associated with greater liking of dark chocolate with effect estimates similar to those for liking coffee without sugar. The *ADORA2A* rs5751876 C variant previously associated with caffeine-induced anxiety and wake promotion was nominally inversely associated with all coffee-liking traits (β ~ − 0.02, 0.002 < P < 0.05) and more strongly and significantly with less liking of dark chocolate (β = − 0.11, P < 0.0001). The same pattern of results was observed for the correlated *ADORA2A* rs5760444 C variant (r^2^ = 0.90, EUR) previously linked to coffee intake in GWAS of Asians^17^. *ADORA2A* (rs2330783 G) was also significantly associated with higher dark chocolate intake and lower beer intake. No other behavior-loci were associated with dark chocolate *intake* in UKB. In post-hoc analysis we examined SNP associations with milk chocolate in UKB and observed statistically significant associations of *ADORA2A* (rs2330783 G), *AHR*, *CYP1A2*, and *POR* with liking milk chocolate, but not milk chocolate intake*.* The directions of associations with liking milk chocolate were opposite to those reported for liking dark chocolate. None of the loci was associated with liking of bitter foods.

Few associations of the evaluated genetic variants with coffee intake met statistical significance (P < 0.002) in US cohorts (Supplementary Table S9). Variants near *AHR* (rs4410790 C) and *CYP1A2* (rs2472297 T) were significantly associated with higher total coffee intake and effect sizes tended to be larger for black, unsweetened, or no-milk coffee than for coffee with added milk or sugar. *CYP1A2* (rs2472297 T) was significantly associated with caffeinated but not decaffeinated coffee. A similar but smaller difference in association between caffeinated and decaffeinated coffee was observed for the *AHR* variant. The odds ratio (95% CI) of reporting ‘avoiding coffee because of its bitterness’ for each additional allele was 0.83 (0.66, 0.99) for *CYP2A6* (rs56113850 C), 0.64 (0.25, 1.03) for *ADORA2A* (rs2330783 G), and 1.13 (1.01, 1.25) for *ADORA2A* (rs5751876 C).

### Genome-wide analysis

Table 4 presents *novel* genome-wide significant (P < 5 × 10^–8^) loci based on a GWAS leveraging the new and more refined coffee and tea phenotypes in UKB (Supplementary Fig. S1, see details in Supplementary Table S10 and S11). Low (λ = 1.04) to moderate (λ = 1.15) genomic inflation was observed across traits.

**Table 4:**
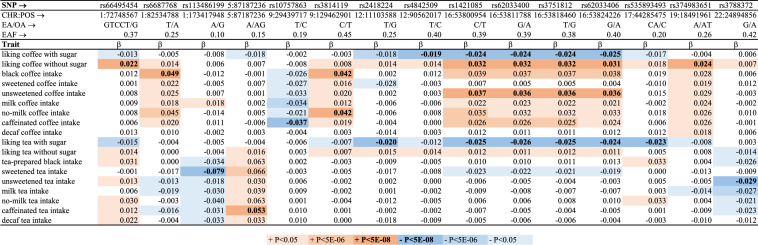
Genome-wide associations of coffee and tea traits in UKB.

Shown are β-coefficients from linear regressions between SNPs (independent variable) and traits (dependent variable). All SNPs present with at least one genome-wide significant (P < 5 × 10^–8^) association in UKB.

*EA* effect allele, *EAF* effect allele frequency, *OA* other allele.

Only associations of new variants at 22q11.23 (near *ADORA2A,* Supplementary Fig. S2) and 12p13 (*TAS2R-*locus, Supplementary Fig. S3) were genome-wide significant in UKB and were also nominally (0.01 < P < 0.04) associated with coffee and tea traits in the US cohorts (Supplementary Table S12). *ADORA2A* (rs3788372 G) was associated with lower unsweetened tea intake in the UKB (P = 2.3 × 10^–8^) and US cohorts (P = 0.02). *TAS2R* (rs2418224 G) was associated with greater liking of tea with sugar (P = 1.6 × 10^–8^) and coffee with sugar (P = 4.3 × 10^–6^) and less liking of tea without sugar (P = 0.0003) and coffee without sugar (P = 0.001) in the UKB. The same variant was nominally associated with liking sweet foods (β = 0.02, P = 0.02) but not bitter foods, dark chocolate or beer (results not shown). While not associated with consumption behavior in UKB, this *TAS2R* variant was associated with lower intakes of black (β = − 0.11, P = 0.02), unsweetened (β = − 0.09, P = 0.03), and no milk (β = − 0.10, P = 0.02) coffee in the US cohorts.

## Discussion

The current study aimed to gain causal insight to the role that taste plays in coffee drinking behavior. Genetically inferred caffeine and bitter taste perception contributed to coffee drinking behavior but, contrary to our hypothesis, to a weaker extent than genetically inferred caffeine sensitivity. Specifically, a greater preference for caffeine inferred by genetic differences in the physiological effects of caffeine leads to a stronger preference for the taste/smell of coffee inferred by liking-scales and reported intake. Similar findings were reported for tea but also dark chocolate.

We examined genetic variants that have previously been associated with bitter taste perception in relation to coffee and tea related traits. In previous GWAS, variants in the *TAS2R* gene have been associated with caffeine (rs2708377 C) and quinine (rs10772420 A) perception; explaining about 2% and 6% of the phenotype variance, respectively^23,25^. The quinine-taste sensitive variant (rs10772420 A) has also been associated with caffeine perception but the effect is weaker and in the opposite direction to that for quinine^25^. We previously examined taste-related variants in relation to total coffee and tea intake in UKB. In that analysis, the quinine-taste sensitive *TAS2R* variant was associated with lower coffee intake and higher tea intake, whereas the caffeine-taste sensitive *TAS2R* variant was associated with higher coffee intake and lower tea intake^19,21^. We now extend that genetic research by using data on food liking and intake of different types of coffee and tea available for a subset of UKB participants. We show that the caffeine-taste sensitive variant (rs2708377 C) is associated with consumption of coffee regardless of how it is prepared but tends to be more strongly associated with caffeinated than with decaffeinated coffee. This result reiterates that caffeine-learned behavior (i.e., experience with its post-ingestive effects), may explain the preference for this naturally bitter tasting chemical^21,51,52^. Coffee, tea, and chocolate do not contain quinine. We observed significant to nominal associations between several of our novel traits and quinine-taste variation (rs10772420) but in directions difficult to interpret. The opposing effects of this variant on quinine and caffeine perception^25^ or complex bitter-sweet taste interactions^53^ may underlie the unusual pattern of associations but merits further study. The well-studied PROP-sensitive *TAS2R38* variants were associated with lower coffee and higher tea intake as we reported previously^19,21^. The direct association between these PROP-sensitive variants and tea consumption was especially strong for sweetened tea suggesting a bitterness-threshold at which tea becomes unpleasant tasting for individuals with these variants. The specificity of these associations with coffee/tea intake (vs. coffee/tea liking), which was not observed for rs2708377 and rs10772420, may be due to chance or perhaps a latent trait distinguishing intake from hedonic traits^54,55^.

Our GWAS identified an additional independent and novel *TASR2* variant in the 12p13.2 region (rs2418224 G) that was associated with greater liking of tea with sugar. The nominally significant and opposite directions of effect on liking sweetened and non-sweetened versions of the beverages, the lack of association with other bitter taste traits, and the association with liking sweet foods, together suggest an effect of this SNP on general *sweet*-perception or preference. Our GWAS analysis in UKB also pointed to *FTO*, a well-established obesity locus. An *FTO* variant (i.e., rs1421085 C), previously associated with higher BMI, was associated with higher unsweetened coffee intake and liking and less liking of sweetened coffee and tea. In our previous GWAS of bitter and sweet beverages the same *FTO* variant was associated with higher coffee intake in the UKB but this result was not replicated in US cohorts^19^. However, the same variant was associated with lower SSB intake in both the UKB and US cohorts. A recent GWAS in UKB also observed associations between *FTO* variants and total sugar intake^56^. Taken together, our current study findings are likely attributed to sweet taste as opposed to coffee or bitter taste and the inability to replicate UKB *FTO*-coffee associations in US cohorts may be due to population differences in the social or food environment. Variants in *MC4R*, *SEC16B* and *TMEM18* that associated with coffee consumption in previous GWAS are also GWAS-confirmed obesity loci. In the current study, the coffee-increasing variants (also the obesity-increasing variants) were inversely associated with liking sweetened coffee and tea. None of these loci were associated with SSB or sweet taste perception in GWAS^19,56^.

Most SNPs identified in previous GWAS of coffee consumption were replicated in the UKB subsample used in the current study. Replicated SNPs were generally more associated with liking coffee without sugar than coffee with sugar but their association with increased coffee *intake* did not vary substantially by preparation type. The stronger association with liking coffee without sugar was unexpected for loci with known roles in caffeine metabolism or physiological effects, but not in taste perception, such as *AHR, CYP1A2, POR, CYP2A6* and *ADORA2A*. Again, these findings suggest that taste/smell and caffeine effects are not as distinct as expected. Individuals consuming more coffee because of a genetic predisposition to increased caffeine metabolism or tolerance may learn to associate coffee/caffeine bitter taste with the favorable physiological effects of caffeine. Our genetic findings align with results of a small clinical study by Masi et al.^57^. Individuals with a higher caffeine metabolism rate (determined by change in salivary caffeine concentrations following intake of caffeine) gave lower bitterness ratings for espresso coffee samples and caffeine, but not quinine, solutions and added less sugar to coffee^57^.

SNPs in *AHR* and *CYP1A2* are the strongest and most robust signals in GWAS of coffee and caffeine intake^11–20^. Consistent with the role of the enzymes encoded by these genes in caffeine metabolism^58^, we observed stronger associations of these variants with caffeinated than with decaffeinated coffee in all cohorts included in the current study. We also observed that *OR5M7P* (rs597045 A) was more strongly associated with caffeinated than decaffeinated coffee in UKB. This variant is not associated with caffeine perception in GWAS^23,25^. *OR5M7P* is a pseudogene upstream of *OR5M8,* one of many genes encoding specific olfactory receptors which function in the perception of smell. Smell and taste are highly related but why this variant affects caffeine-and not taste-related traits is unclear but may be another case in-point of conditioned taste preferences.

We investigated three SNPs in *ADORA2A*, encoding the adenosine 2A receptor, a target for caffeine which mediates the psychostimulant effect of the drug^58^. As such, we did not expect SNPs in *ADORA2A* to be differentially associated with coffee and tea traits defined independent of caffeine content. *ADORA2A* (rs5751876 C) is thought to increase sensitivity to caffeine as it has been associated with greater caffeine-induced anxiety and alertness and lower caffeine intake in several candidate gene studies^45,58,59^. In the current study, this variant was associated with less liking of coffee (with or without sugar) and while not associated with coffee consumption it was associated with higher tea intake in the UKB. Taken together, these results suggest that individuals with *ADORA2A* (rs5751876 C) avoid heavy caffeine intake. Since coffee contains twice the amount of caffeine than tea, these individuals may prefer tea over coffee. In US cohorts, rs5751876 C carriers were more likely to avoid coffee because it tastes bitter; a finding that further illustrates how some individuals are unable to separate the physiological effects of caffeine from taste preferences. *ADORA2A* (rs2330783 G) was associated with liking coffee without sugar and higher coffee intake without differences in association by type of coffee. In US cohorts, rs2330783 G carriers were less likely to avoid coffee because of its bitter taste. Our GWAS of unsweetened tea in UKB identified variants near *ADORA2A* (i.e., rs3788372) not in LD with those described above that was also associated with unsweetened tea in the US cohorts. Unlike *AHR* and *CYP1A2*, none of the SNPs in *ADORA2A* differentially associated with caffeinated and decaffeinated coffee or tea. To our knowledge, adenosine receptors do not play a role in taste perception. Whether our results for *ADORA2A* rs2330783 and rs3788372 are mediated by taste or caffeine is unclear and warrants further investigation.

*ADORA2A, CYP1A2, CYP2A6, AHR* and, to a weaker extent, *POR* variants associated with higher coffee intake (US cohorts) and liking (UKB) were also associated with increased dark chocolate intake (US cohorts) and liking (UKB). No association or associations in the opposite direction were observed with liking and intake of milk chocolate and other bitter foods, suggesting that caffeine (its psychostimulant effects, taste, or both) may be underlying the observed associations with dark chocolate. Dark chocolate contains more caffeine per weight than milk chocolate^60^ and while the amount is still less than the content in coffee and tea it may be detected by individuals sensitive to caffeine. Dark chocolate is also a unique source of theobromine, another methylxanthine with psychostimulant effects^60–63^.

Strengths of the current study include the use of novel and comprehensive coffee and tea traits in independent cohorts. Nevertheless, several limitations need to be considered. The liking and dietary intake measures used are subject to bias and measurement error as discussed in detail previously^64–66^. Specifically, the 24-h diet collections in UKB may not reflect usual intake and the FFQs used in the US cohorts may be prone to reporting errors. In addition, liking traits were only available for UKB and not for the US cohorts. The US cohorts included a non-representative group of elderly whose sense of taste and small may be reduced and beverage choices more likely affected by medical issues. Several genetic variants that were associated with coffee or caffeine intake in previous GWAS studies were not replicated in the current study. That probably reflects the smaller sample size in the sub-cohorts with more detailed information used in the current analysis. In addition, the association between *ALDH2* variation and coffee consumption has only been reported in Japanese^14^ and given the low frequency of these variants in populations of European ancestry the lack of replication in the current study was expected. Finally, there are currently no published GWAS-SNPs for perception of other bitter compounds in coffee such as maillard reaction products, cafestol, chlorogenic acid derivatives or other uncharacterized coffee-bitter compounds^67–69^ thus limiting our candidate SNP-approach.

Genetic markers of coffee and caffeine consumption are increasingly used as instrumental variables to seek causal insight to coffee/caffeine and health^70^. Whether a genetic instrument captures total coffee/caffeine intake, only certain types of coffee, or not only coffee but a broader characteristic, impacts the interpretation and translation of studies. For example, evidence for a causal relationship between *black* coffee and type 2 diabetes is very different than a causal relationship between coffee and type 2 diabetes. A cautionary approach to genetic instrumental variable studies is particularly relevant now that weaker non-genome-wide significant variants are included in such studies.

In summary, our genetic analysis suggests the psychostimulant effects of caffeine outweighs the bitterness of caffeine. A greater preference for caffeine based on genetic differences in the physiological effects of caffeine leads to a stronger preference for the taste/smell of coffee and dark chocolate. Similarly, greater sensitivity to the adverse physiological effects of caffeine was associated with avoiding the taste of coffee. Taste preferences and physiological caffeine effects thus seem to become entangled in a way that is difficult to distinguish for individuals. These potential examples of conditioned taste preferences or aversions merit further clinical investigation. This apparent disruption of an innate aversion to bitter taste and its genetic correlation with coffee preferences has important relevance to food and beverage development as well as genetic epidemiological studies of coffee.

## Supplementary Information

Supplementary Information 1.

Supplementary Information 2.

## Data Availability

Data described in the manuscript is available to all researchers and can be accessed upon approval of the UK Biobank https://www.ukbiobank.ac.uk/enable-your-research/apply-for-access , HPFS https://sites.sph.harvard.edu/hpfs/for-collaborators/ and NHS https://nurseshealthstudy.org/researchers boards.

## Code availability

Statistical code used in this study is available upon reasonable request from the corresponding author.
