# Supplementary material for: Genetic determinants of liking and intake of coffee and other bitter foods and beverages
Source: Sci Rep. 2021 Dec 13;11:23845. doi: 10.1038/s41598-021-03153-7 (PMC8669025; doi:10.1038/s41598-021-03153-7)

Supplementary Information

# **SUPPLEMENTARY METHODS**

## UK Biobank Participants

The targeted population for the UK Biobank included ~9.2 million individuals registered with the UK’s National Health Service who were aged 40–69 years and lived within approximately 40 km of one of 22 assessment centers located throughout England, Wales, and Scotland^1^. Between 2006 and 2010, over 502,633 participants aged 37-73 years (5.4% participation rate) provided full informed consent to participate in UK Biobank and completed a 90-minute assessment that included i) touchscreen questionnaires on sociodemographic factors, lifestyle and medical history ii) an in-person interview and iii) physical assessment. Subsets of the cohort have returned for follow-up assessments and have completed on-line questionnaires.

## Food Preferences

The Food Preferences Questionnaire was first piloted with 10,000 participants to ensure the platform and procedures were adequate and robust in terms of acceptability of content and length. In 2019, all participants with a contact email address were invited to complete the questionnaire. UK Biobank’s re-contact approach was as follows: initial invitation email; a reminder email to non-responders sent 2 weeks after the initial invite; a reminder to partial responders 2 weeks after they started the questionnaire; a final reminder sent to non-responders 3 months after the initial invite. 81.1% of participants completed the questionnaire in less than 20 minutes. Overall, 333,344 participants were sent an email invitation, of whom 181,224 (54.4%) fully completed the questionnaire. The first and last questionnaire were completed May 2019 and January 2020, respectively. A further 996 participants accessed the questionnaire via the participant website without having received an email invite (because they have not provided UK Biobank with a valid email address or completed the questionnaire via the participant website prior to an invite being sent).


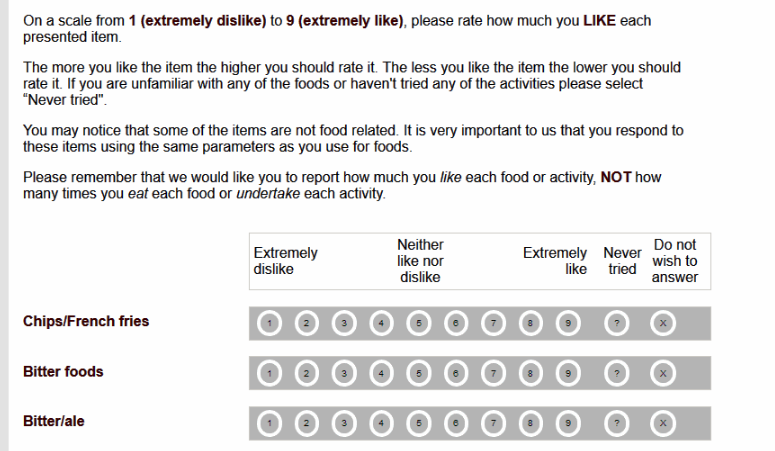


## Online 24 hour dietary recalls

Dietary intake was also collected using a 24-hour recall questionnaire (Oxford WebQ) in a subset of participants ^2^. We used the mean intake from participants who completed at least two of the five dietary recalls. Screenshots of questionnaire items pertaining to coffee and tea intake are below. Categorical measures of coffee/tea quantity were converted to cups/d by using the midpoint of each category; those reporting 6+ cups/d were assigned an intake of 6 cups/d. The question concerning milk/cream/whitener was asked for each of i) instant ii) filter/americano/cafetiere iii) espresso and iv) other coffee drinks. The question concerning decaffeinated tea was only asked for standard tea. The addition of milk to tea question was asked for each of i) standard and ii) rooibos/redbush tea. For participants responding “varied” to decaffeinated questions we coded half their reported coffee (or tea) intake as regular and the other half as decaffeinated. We recoded “varied” responses for sweeteners to i) the quantity specified in another diet record of the same individual or, if not available or also “varied”, to ii) the median response of the full sample for the beverage in question: 1 tsp for both coffee and tea. We estimated total sugar (or artificial sweetener) added to coffee (or tea) per day but multiplying the quantity of sugar (or artificial sweetener) added by the number of cups of coffee (or tea) consumed. Given the non-quantifiable nature of the milk added question, we recoded “varied” to “yes”.


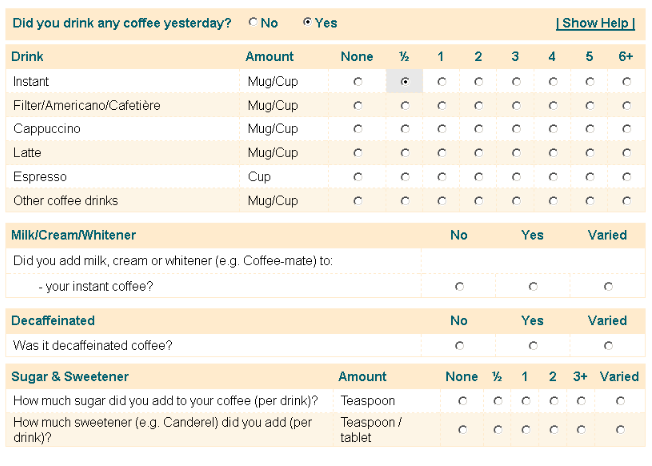


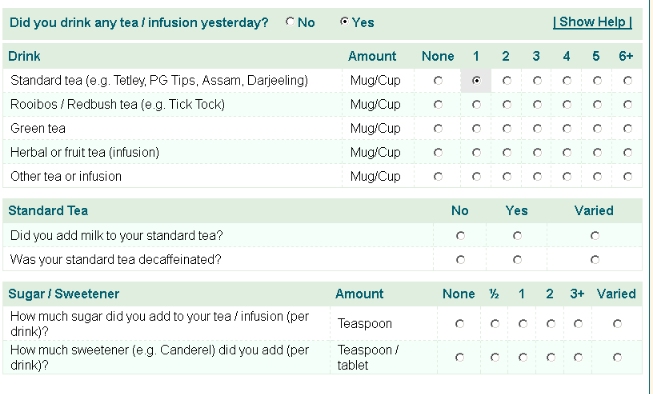


**References**

1. UK Biobank Coordinating Centre. UK Biobank: Protocol for a large-scale prospective epidemiological resource. (ed^(eds) (2007).

2. Galante J*, et al.* The acceptability of repeat Internet-based hybrid diet assessment of previous 24-h dietary intake: administration of the Oxford WebQ in UK Biobank. *British Journal of Nutrition* **115**, 681-686 (2016).

3. Coltell O*, et al.* Association between taste perception and adiposity in overweight or obese older subjects with metabolic syndrome and identification of novel taste-related genes. *Am J Clin Nutr* **109**, 1709-1723 (2019).

4. Ledda M*, et al.* GWAS of Human Bitter Taste Perception Identifies New Loci and Reveals Additional Complexity of Bitter Taste Genetics. *Hum Mol Genet* **23**, 259-267 (2013).

5. Reed DR*, et al.* The perception of quinine taste intensity is associated with common genetic variants in a bitter receptor cluster on chromosome 12. *Hum Mol Genet* **19**, 4278-4285 (2010).

6. Hwang L-D*, et al.* Bivariate genome-wide association analysis strengthens the role of bitter receptor clusters on chromosomes 7 and 12 in human bitter taste. *BMC genomics* **19**, 678 (2018).

7. Zhong V*, et al.* A genome-wide association study of habitual bitter and sweet beverage consumption. *Submitted*, (2018).

8. Matoba N*, et al.* GWAS of 165,084 Japanese individuals identified nine loci associated with dietary habits. *Nature human behaviour* **4**, 308-316 (2020).

9. Cornelis MC*, et al.* Genome-wide meta-analysis identifies six novel loci associated with habitual coffee consumption. *Molecular psychiatry* **20**, 647 (2015).

10. Pirastu N*, et al.* Non-additive genome-wide association scan reveals a new gene associated with habitual coffee consumption. *Scientific reports* **6**, 31590 (2016).

11. Sulem P*, et al.* Sequence variants at CYP1A1-CYP1A2 and AHR associate with coffee consumption. *Hum Mol Genet* **20**, 2071-2077 (2011).

12. Nakagawa-Senda H*, et al.* A genome-wide association study in the Japanese population identifies the 12q24 locus for habitual coffee consumption: The J-MICC Study. *Scientific reports* **8**, 1493 (2018).

13. Jia H*, et al.* GWAS of habitual coffee consumption reveals a sex difference in the genetic effect of the 12q24 locus in the Japanese population. *BMC Genet* **20**, 61 (2019).

14. Cornelis MC*, et al.* Genome-wide meta-analysis identifies regions on 7p21 (AHR) and 15q24 (CYP1A2) as determinants of habitual caffeine consumption. *PLoS genetics* **7**, e1002033 (2011).

15. Zhong VW*, et al.* A genome-wide association study of bitter and sweet beverage consumption. *Human molecular genetics*, (2019).

16. Amin N*, et al.* Genome-wide association analysis of coffee drinking suggests association with CYP1A1/CYP1A2 and NRCAM. *Molecular psychiatry* **17**, 1116-1129 (2012).

17. Pirastu N*, et al.* A Genome-Wide Association Study in isolated populations reveals new genes associated to common food likings. *Reviews in Endocrine and Metabolic Disorders* **17**, 209-219 (2016).

18. Furukawa K*, et al.* A Genome-Wide Association Study Identifies the Association between the 12q24 Locus and Black Tea Consumption in Japanese Populations. *Nutrients* **12**, (2020).

19. Cornelis MC*, et al.* Genome-wide association study of caffeine metabolites provides new insights to caffeine metabolism and dietary caffeine-consumption behavior. *Hum Mol Genet* **25**, 5472-5482 (2016).

20. Retey JV*, et al.* A genetic variation in the adenosine A2A receptor gene (ADORA2A) contributes to individual sensitivity to caffeine effects on sleep. *Clin Pharmacol Ther* **81**, 692-698 (2007).

21. Byrne EM*, et al.* A genome-wide association study of caffeine-related sleep disturbance: confirmation of a role for a common variant in the adenosine receptor. *Sleep* **35**, 967-975 (2012).

22. Cornelis MC, El-Sohemy A, Campos H. Genetic polymorphism of the adenosine A2A receptor is associated with habitual caffeine consumption. *Am J Clin Nutr* **86**, 240-244 (2007).

23. Alsene K, Deckert J, Sand P, de Wit H. Association between A2a receptor gene polymorphisms and caffeine-induced anxiety. *Neuropsychopharmacology* **28**, 1694-1702 (2003).

24. Hindorf L*, et al.* Catalogue of Published Genome-Wide Association Studies. (ed^(eds) (accessed January 1, 2015).

# Table S1. GWAS confirmed SNPs for coffee-, tea- and caffeine- related traits*

| Locus | Closest gene(s) | SNP, EA | EAF | | | | Race  Origin | Trait | Effect | Reference | Assoc. with other traits (GWAS) |
| --- | --- | --- | --- | --- | --- | --- | --- | --- | --- | --- | --- |
|  |  |  | AFR | AMR | ASN | EU |  |  |  |  |  |
| Taste-Loci | | | | | | | | | | | |
| 7q34 | *TAS2R38* | rs713598,G  missense  C145G:A49P  rs1726866, G  missense A785G, V262A  (haplotype)  rs10246939, C  missense T886C:I296V | 0.51  0.69  0.52 | 0.71  0.66  0.64 | 0.65  0.65  0.65 | 0.42  0.45  0.45 | EU/brazil | PROP/PTC perception | + | ^3-6^ | PAV/PAV: tasters |
| 12p13.2 | *TAS2R19* | rs10772420, A  missense | 0.39 | 0.53 | 0.44 | 0.60 | EU | quinine perception (sensitivity) | + | ^6^ | Caffeine perception (weaker and opposite direction of effect) |
|  | *PRH1*  *candidate: TAS2R14* | rs2708377,C  (other T) | 0.59 | 0.15 | 0.06 | 0.13 | Brazil | caffeine perception  (sensitivity) | + | ^4^ | LD with rs319277 (r^2^=0.95), associated with decreased tea consumption in UKB but not replicated in US cohorts^7^ |
|  |  | rs2597979,G  (LD with above) | 0.58 | 0.20 | 0.10 | 0.16 | EU | caffeine perception  (sensitivity) | + | ^6^ |  |
| Behavior-Loci | | | | | | | | | | | |
| 1q21.3 | *MCL1,ENSA* | rs6681426, G | 0.33 | 0.43 | 0.34 | 0.35 | ASN | coffee intake | + | ^8^ | Replicated in UKB for coffee, tea  Lung function, |
| 1q25.2 | *SEC16B* | rs574367, T  intergenic | 0.09 | 0.15 | 0.19 | 0.21 | EU | coffee intake | + | ^7^ | BMI, obesity, menarche  (LD: waist/hip circumference, weight, adiposity measures, menarche, T2D, height) |
| 2p25.3 | *TMEM18* | rs10865548, G  intergenic | 0.93 | 0.85 | 0.9 | 0.83 | EU | coffee intake | + | ^7^ | BMI, obesity, weight, menarche  (LD: T2D, smoking status, CRP, uric acid, hand-grip strength, adiposity measures, |
| 2p23.3 | *GCKR* | rs1260326, C  missense | 0.88 | 0.59 | 0.44 | 0.59 | EU/AFR/ASN | coffee intake | + | ^7-9^ | Alcohol intake, bitter alcoholic beverage intake, total bitter beverage intake, waist circumference, lipid traits, serum glucose, chronic kidney disease, C-reactive protein, gamma-glutamyl transferase, serum albumin, serum urate, leptin |
| 4q22.1 | *ABCG2* | rs1481012, A  intronic | 0.98 | 0.85 | 0.71 | 0.89 | EU/AA/ASN | coffee intake | + | ^8, 9^ | Total bitter beverage intake, LDL response to statin, serum urate, gout |
| 6q21 | *PDSS2* | rs2216084,T  intronic | 0.96 | 0.64 | 0.99 | 0.67 | EU | coffee intake | + | ^10^ |  |
| 7p21.1 | *AHR* | rs4410790,C  intergenic | 0.47 | 0.42 | 0.37 | 0.62 | EU/AA/ASN | coffee intake | + | ^7-9, 11-13^ | Bitter non-alcoholic beverage intake, total bitter beverage intake, albuminuria, plasma caffeine |
|  |  |  |  |  |  |  | EU | caffeine intake | + | ^14^ |  |
|  |  |  |  |  |  |  | EU/ASN | tea intake | + | ^8, 15^ |  |
| 7q11.23 | *MLXIPL* | rs7800944,C  intronic | 0.39 | 0.18 | 0.11 | 0.27 | EU/ASN | coffee intake | + | ^7-9^ | Bitter non-alcoholic beverage intake, lipid traits, alcohol drinking, fasting glucose‡ |
| 7q11.23 | *POR* | rs17685, A  3’UTR | 0.13 | 0.22 | 0.35 | 0.30 | EU/AA/ASN | coffee intake | + | ^7-9^ | Bitter non-alcoholic beverage intake, total bitter beverage intake |
| 7q31.1 | *NRCAM* | rs382140, A  intergenic | 0.45 | 0.21 | 0.19 | 0.18 | EU | coffee intake | + | ^16^ |  |
| 11p12 | *BDNF* | rs6265, C |  |  |  |  | EU | coffee intake | + | ^8, 9^ |  |
| 11p14.2 | *FIBIN* | rs12274052, A  [proxy (r^2^=0.7) for  rs145671205, T ]  intergenic | 0.73  0.96 | 0.91  0.93 | 0.91  0.94 | 0.92  0.93 | EU | coffee liking | + | ^17^ |  |
| 11q12.1 | *OR5M7P* | rs597045, A  intergenic | 0.97 | 0.82 | 0.86 | 0.69 | EU | coffee intake | + | ^7^ |  |
| 12q24.11-13 | *ALDH2* | rs671, A  (other A)  missense | 0 | 0 | 0.12 | 0 | ASN | coffee intake  green tea intake | + | ^8, 12, 13^ | AD, AD symptoms, alcohol intake,  flushing response lipid traits, , gamma-glutamyl transferase, serum urate, serum alpha-1-antitrypsin, esophageal cancer, hemoglobin, CHD, brain aneurysm, metabolic syndrome, BMI, sweet preference, black tea intake |
|  | *HECTD4* | rs2074356, T  intronic  (other C) | 0 | 0 | 0.16 | 1 | ASN | black tea intake | + | ^18^ | Alcohol intake, esophageal cancer, gamma-glutamyl transpeptidase, HDL cholesterol, renal function traits, glucose levels, |
| 14q12 | *AKAP6* | rs1956218, G  intronic | 0.68 | 0.66 | 0.44 | 0.53 | EU | coffee intake | + | ^7^ |  |
| 15q24.1 | *CYP1A1-CYP1A2* | rs2472297, T  intergenic | 0.02 | 0.09 | 0.0 | 0.24 | EU/AA/ASN | coffee intake  (tea) | + | ^7-9, 11, 16^ | Bitter non-alcoholic beverage intake, total bitter beverage intake, albuminuria, urine albumin, plasma caffeine |
|  |  |  |  |  |  |  |  | caffeine intake | + | ^14^ |  |
|  | *CYP1A2* | rs762551  A/C  intronic | 0.54 | 0.74 | 0.63 | 0.69 | EU | coffee intake | + | ^9^ |  |
|  |  |  |  |  |  |  |  | caffeine intake | + | ^9^ |  |
| 17q11.2 | *EFCAB5, NSRP1, SLC6A4* | rs9902453, G  intronic | 0.08 | 0.52 | 0.71 | 0.45 | EU/AA | coffee intake | + | ^9^ |  |
| 18q21.32 | *MC4R* | rs66723169, A  intergenic | 0.11 | 0.13 | 0.2 | 0.23 | EU | coffee intake | + | ^7^ | BMI, obesity, height, |
| 19q13.2 | *CYP2A6* | rs56113850, C |  |  |  |  |  | Plasma paraxanthine/caffeine ratio  (nominally associated with coffee intake in UKB) | -  (+) | ^19^ | Smoking behaviors |
| 22q11.23 | *SPECC1L-ADORA2A* | rs2330783, G  intronic | 0.92 | 0.98 | 0 | 0.99 | EU | coffee intake (tea) | + | ^7^ |  |
|  | *ADORA2A* | rs5751876, C  intronic | 0.34 | 0.57 | 0.51 | 0.61 |  | caffeine intake  caffeine-induced anxiety and wakefulness | -  + | ^20-23^ |  |
|  |  | rs5760444,C | 0.28 | 0.53 | 0.56 | 0.59 | ASN | coffee intake | - | ^8^ | Replicated in UKB but for tea. |

EA: effect allele, EAF: effect allele frequency (based on HapMap data), OA: other allele, AFR: African ancestry, AMR: Ad-mixed American ancestry, ASN: Asian ancestry; EU European ancestry

*Listed are SNPs examined in the current study identified by genome-wide meta-analysis or GWAS discovery/replication designs. rs5751876 (*ADORA2A*) is a candidate SNP selected from the literature. See main Methods for SNP-selection criteria.

†Other GWAS catalogue traits associated with SNP^24^

# Table S2. Characteristics by tea drinking status*

| Variable | UK Biobank | | NHS | | HPFS | |
| --- | --- | --- | --- | --- | --- | --- |
|  | Non-Drinkers n=11473 | Drinkers n=74533 | Non-Drinkers n=575 | Drinkers n=2709 | Non-Drinkers n=575 | Drinkers n=1088 |
| Age at diet collection, years | 57.4±8.0 | 58.3±7.7 | 82.1±6.2 | 81.4±5.9 | 81.5±6.3 | 81.3±6.6 |
| Age at liking/preference collection, years | 65.7±7.7 | 66.6±7.5 | 74.4±6.2 | 73.8±5.9 | 77.6±6.3 | 77.3±6.6 |
| Female, n (%) | 5931 (52) | 41935 (56) | 575 (100) | 2709 (100) | 0 (0) | 0 (0) |
| Current Smoker, n (%) | 1126 (10) | 4603 (6) | 27 (5) | 67 (3) | 8 (1) | 8 (1) |
| BMI, m/kg^2^ | 27.8±5.1 | 26.5±4.5 | 26.6±5.5 | 26.5±5.1 | 26.1±4.0 | 26.0±3.9 |
| BMI ≥30 m/kg^2^, n (%) | 3112 (27) | 13570 (18) | 124 (22) | 571 (21) | 91 (16) | 142 (13) |
| Alcohol intake, g/d | 18.1±21.3 | 16.1±18.2 | 8.4±14.2 | 6.5±10.2 | 14.9±17.4 | 12.9±14.7 |
| Energy intake, Kcal/d | 2006±447 | 2050±430 | 1579±539 | 1690±537 | 1965±574 | 2072±581 |
| Total tea, cups/d | -- | 3.1±1.6 | -- | 1.0±1.2 | -- | 0.8±1.0 |
| Tea preference, n (%) |  |  |  |  |  |  |
| prepared-black | -- | 11344 (15) | -- | 1079 (40) | -- | 507 (47) |
| unsweetened | -- | 60601 (81) | -- | 1595 (59) | -- | 685 (63) |
| no-milk | -- | 12558 (17) | -- | 2209 (82) | -- | 993 (91) |
| sweetened | -- | 13597 (18) | -- | 1377 (51) | -- | 533 (49) |
| milk | -- | 62481 (84) | -- | 763 (28) | -- | 225 (21) |
| Total coffee, cups/d | 3.0±1.7 | 1.6±1.3 | 1.7±1.4 | 1.6±1.3 | 1.7±1.5 | 1.6±1.4 |
| Avoid/Drink less tea because bitter†, n (%) | -- | -- | 65 (14) | 155 (6) | 51 (11) | 38 (4) |
| Dark chocolate, servings/d | 0.04±0.16 | 0.04±0.15 | 0.07±0.19 | 0.09±0.21 | 0.13±0.35 | 0.14±0.27 |
| Beer, servings/d | 0.38±0.80 | 0.30±0.66 | 0.06±0.25 | 0.04±0.19 | 0.28±0.61 | 0.21±0.45 |

*Shown are mean±SD for continuous variables or n (%) for categorical variables. Non-drinkers are defined as consuming no tea. Drinkers are defined as consuming any tea (>0 cups/d).

†Missing data for 173 NHS and 125 HPFS participants.

# Figure S1.

Manhattan Plots for GWAS of coffee and tea traits in UKB

A. Liking coffee with sugar


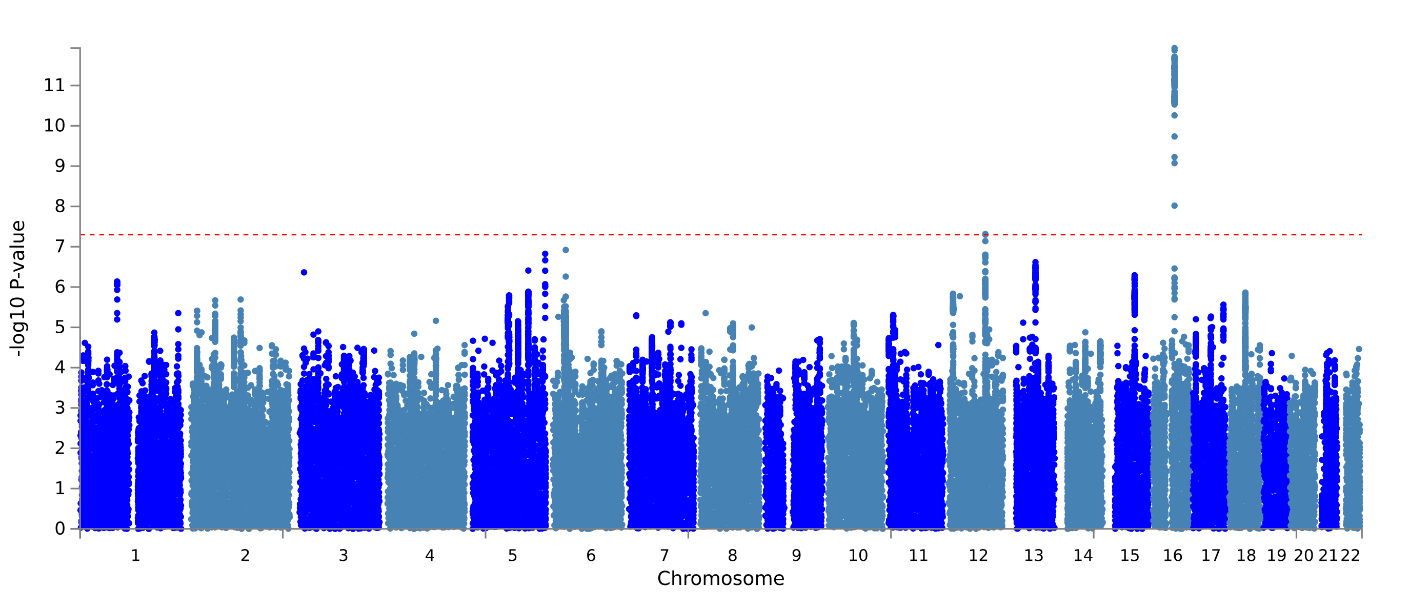


B. Liking coffee without sugar


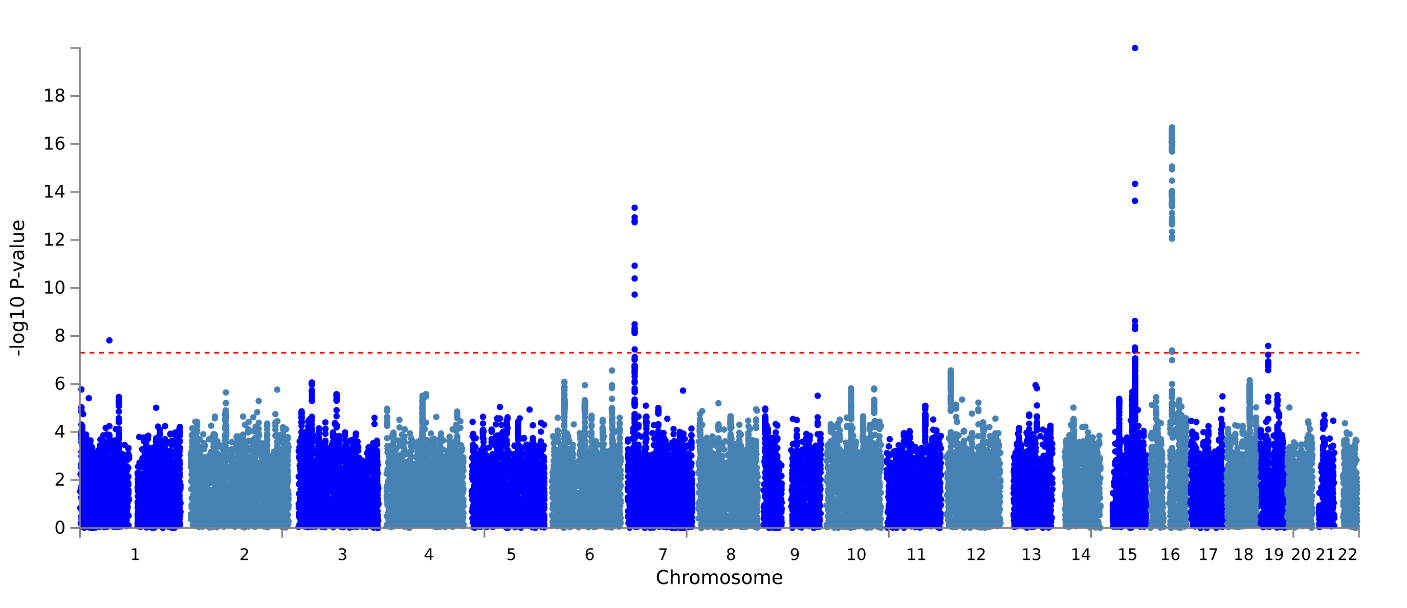


C. Black coffee intake


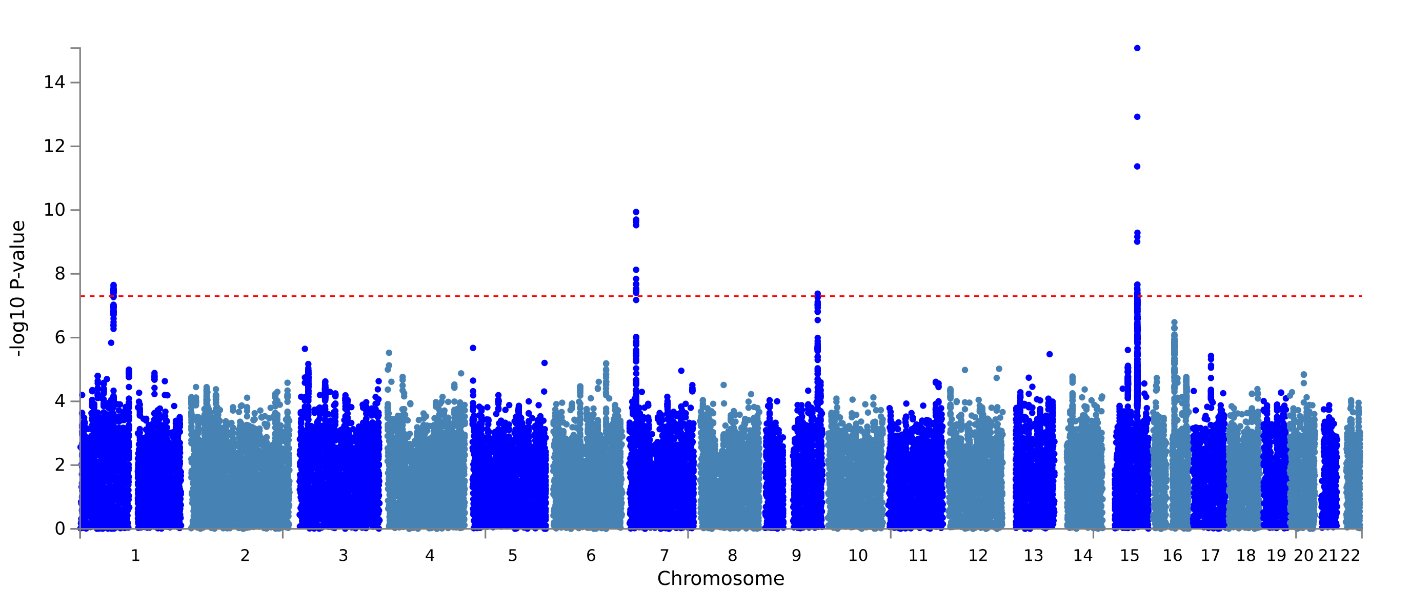


D. Sweetened coffee intake


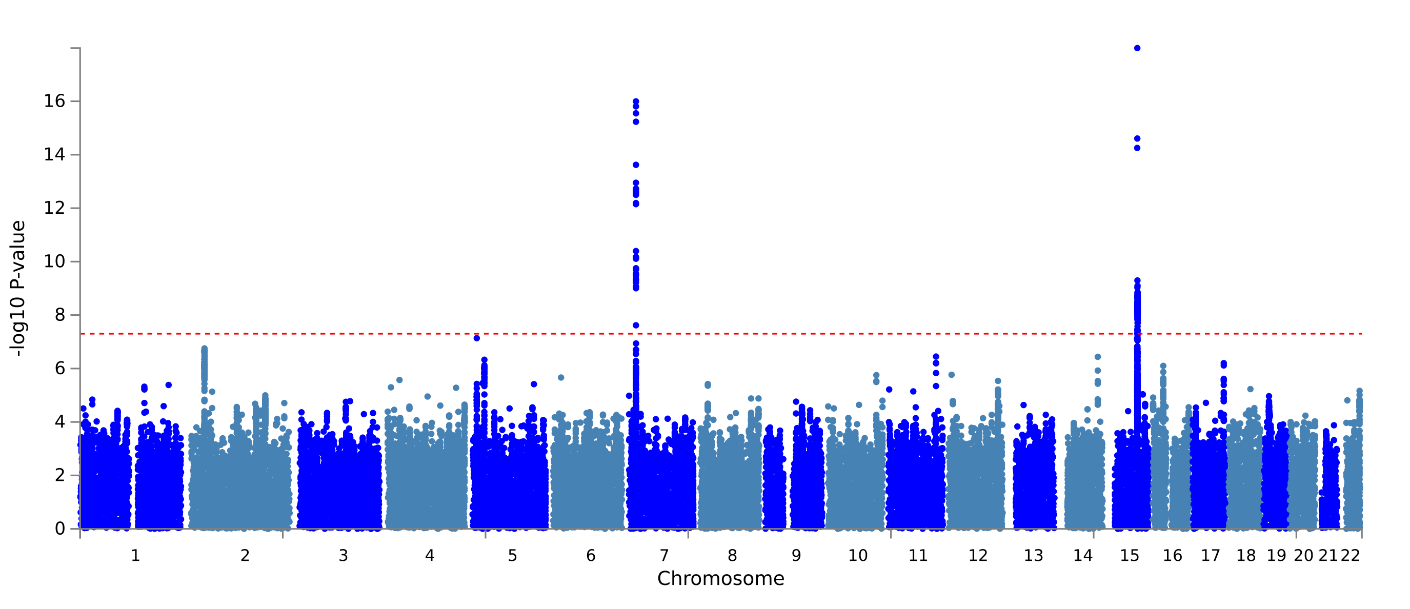


E. Unsweetened coffee intake


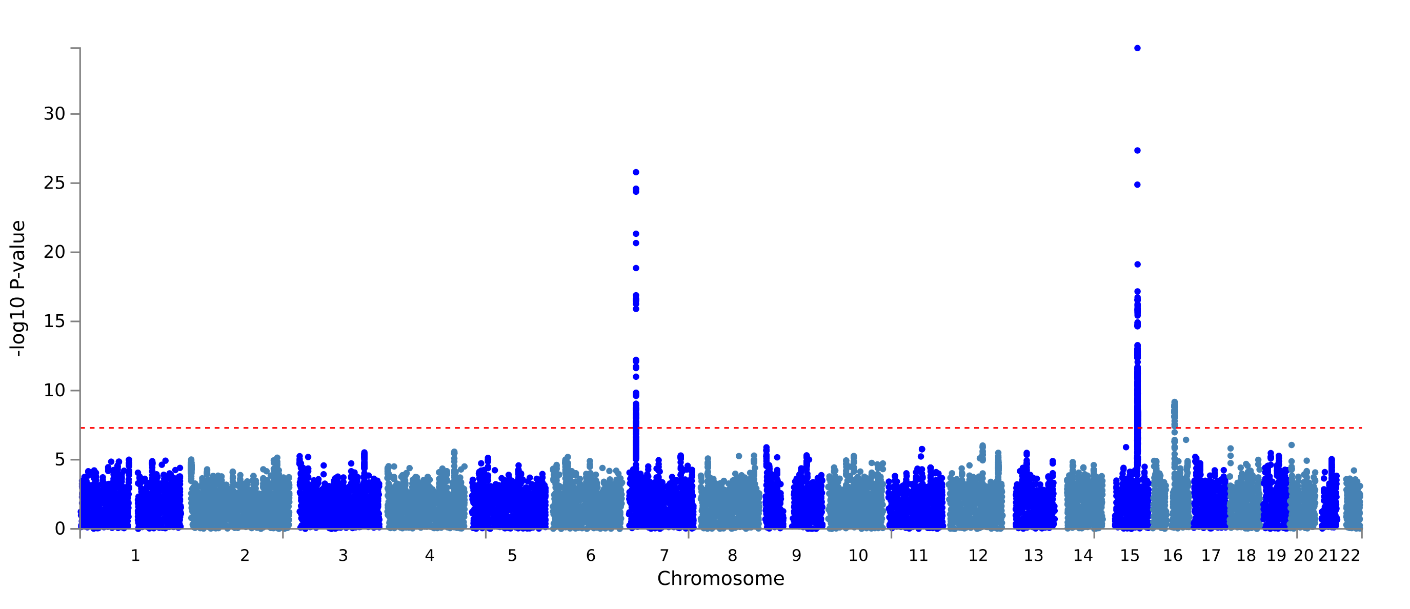


F. Milk coffee intake


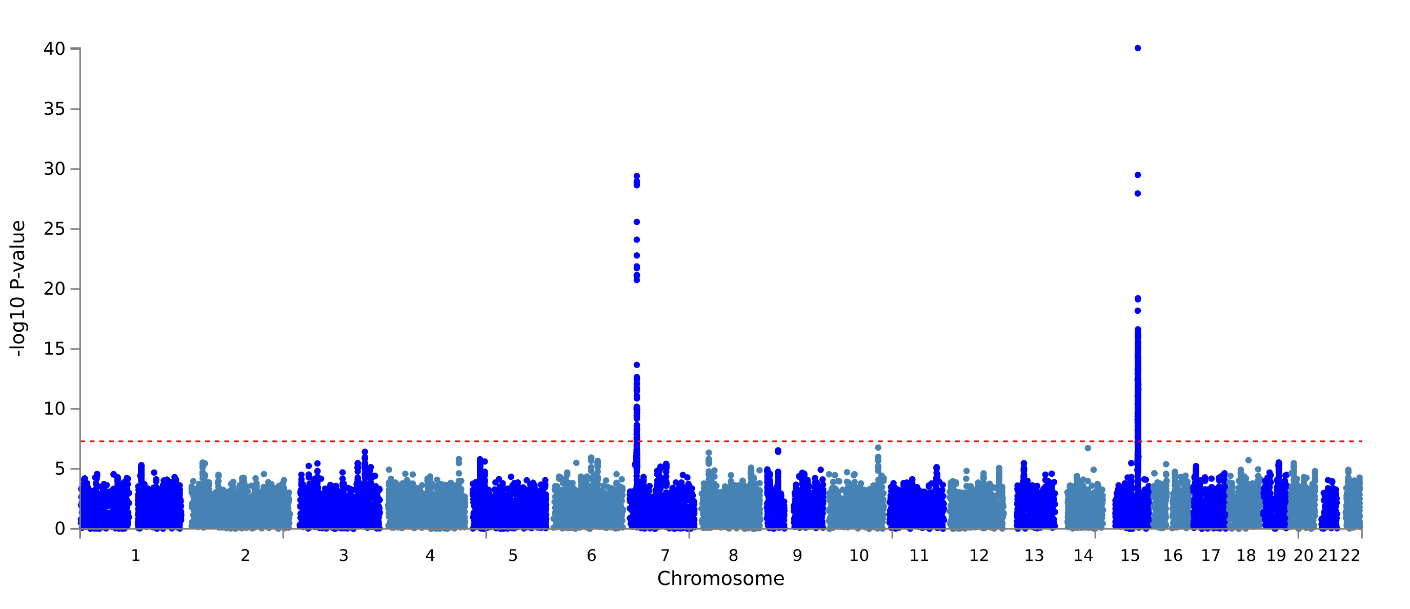


G. No-milk coffee intake


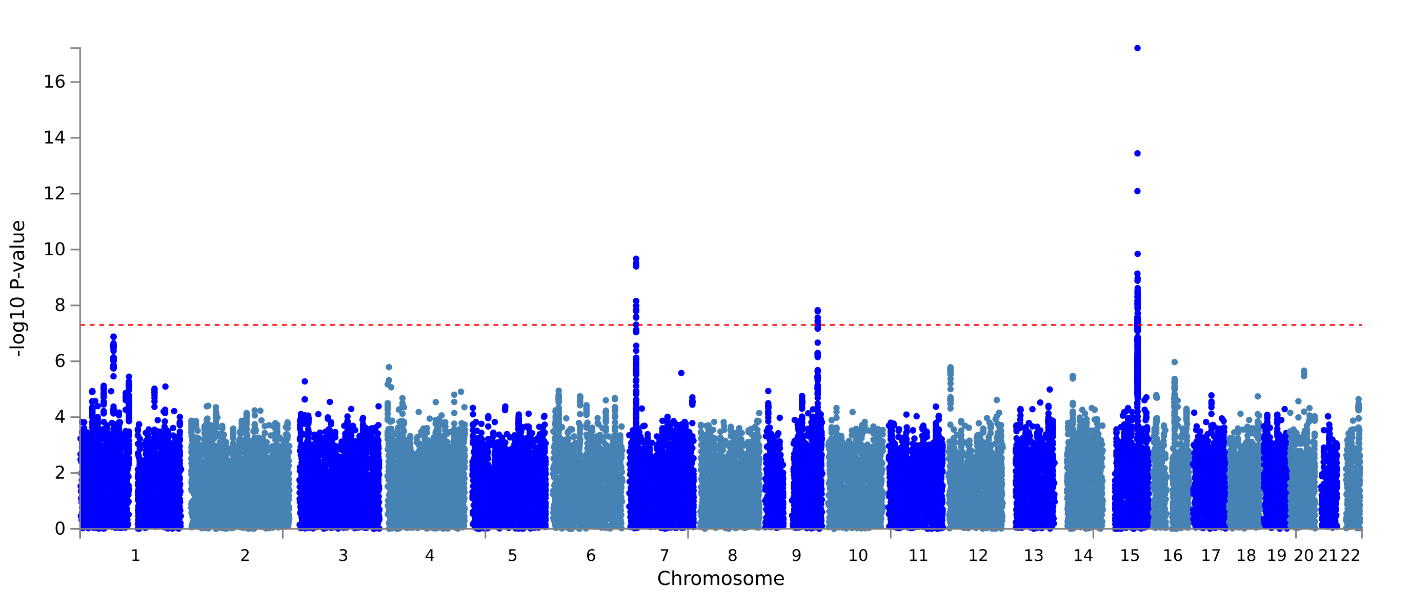


H. Caffeinated coffee intake


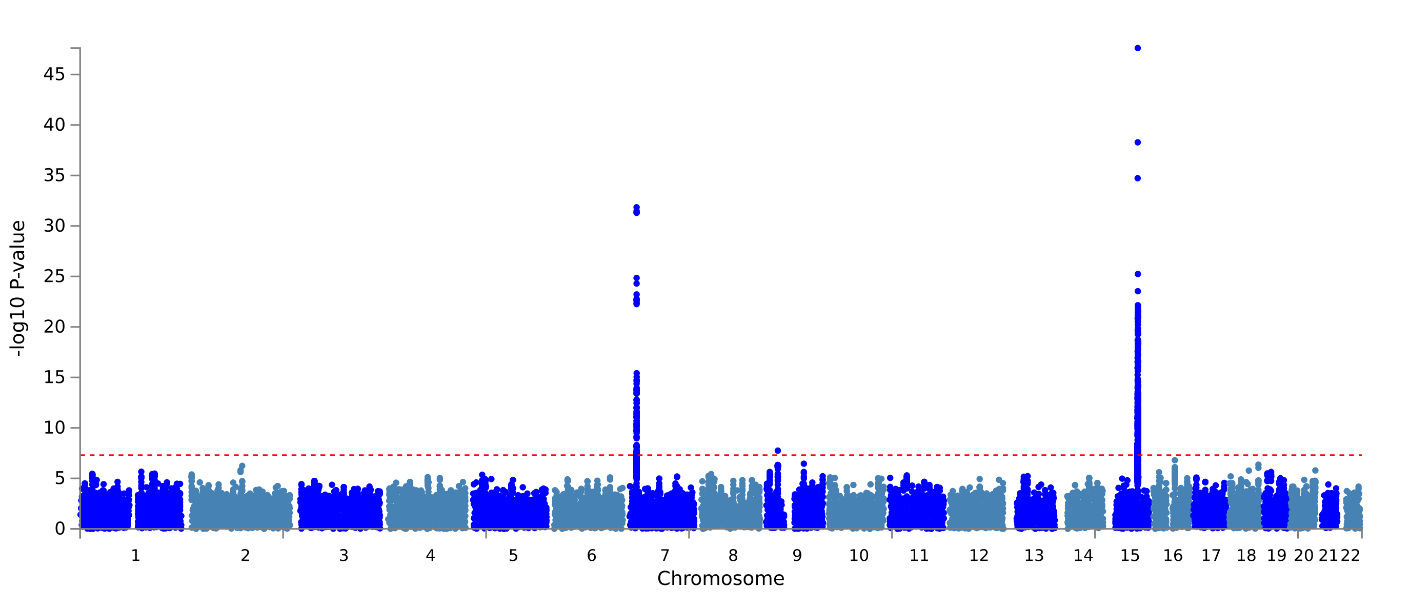


I. Liking tea with sugar


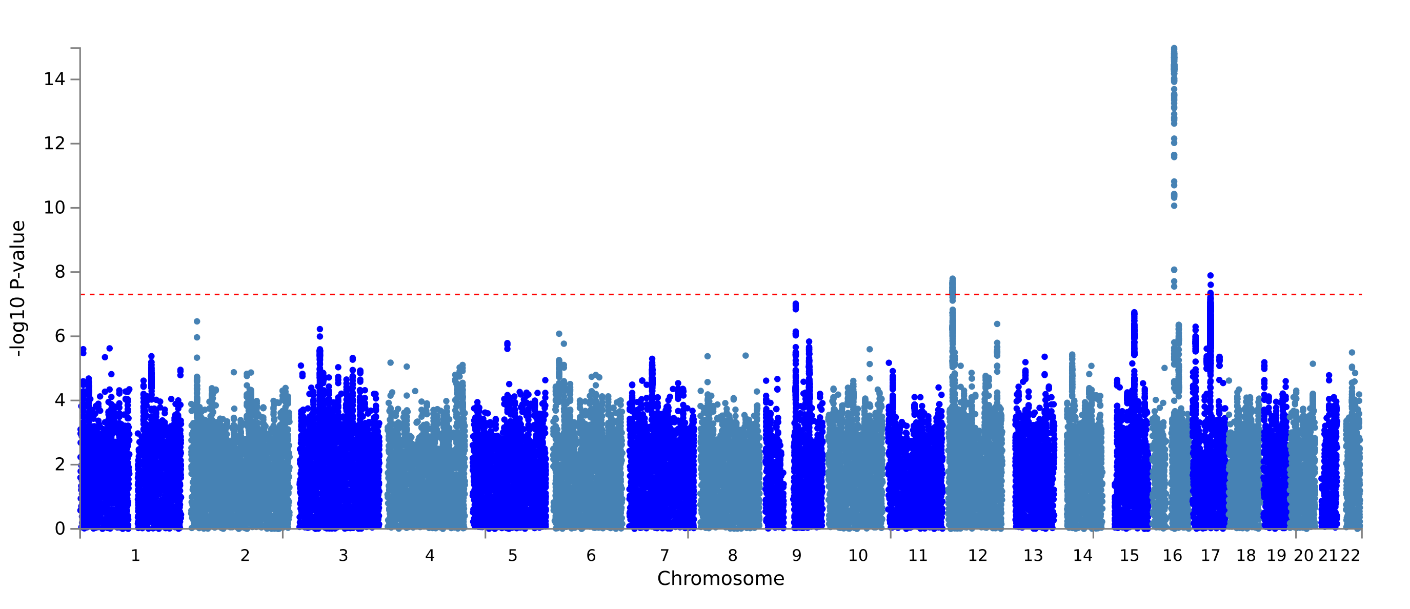


J. Sweetened tea intake


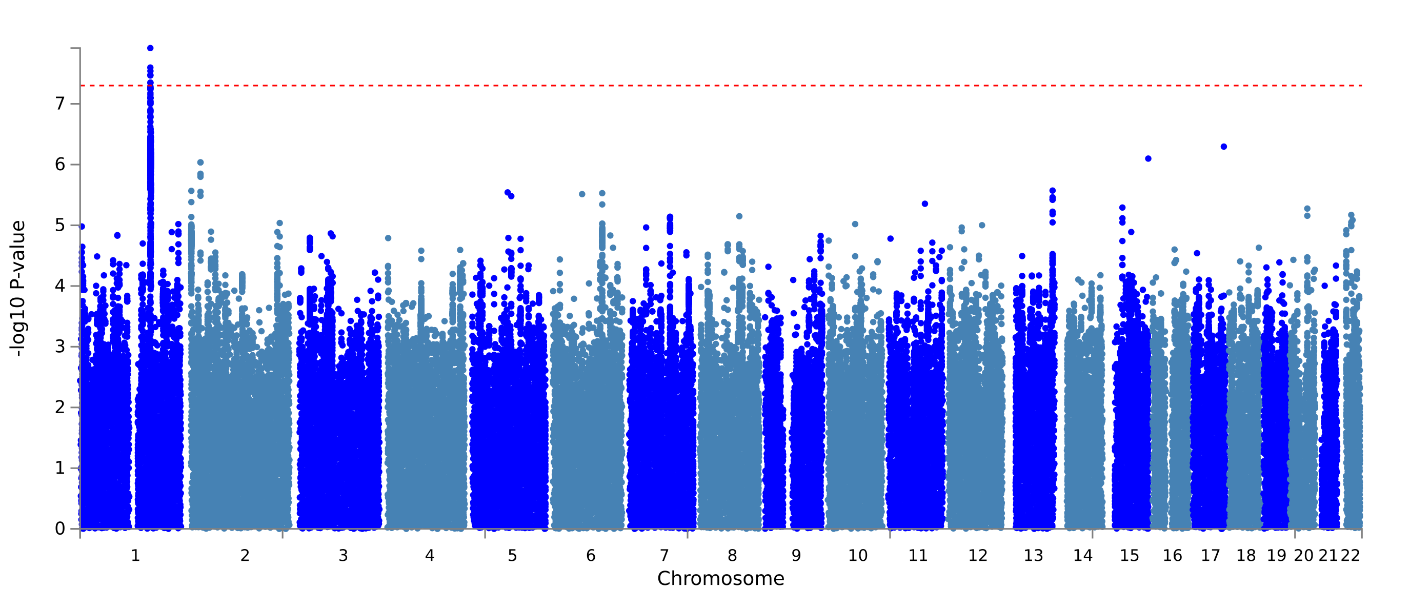


K. Unsweetened tea intake


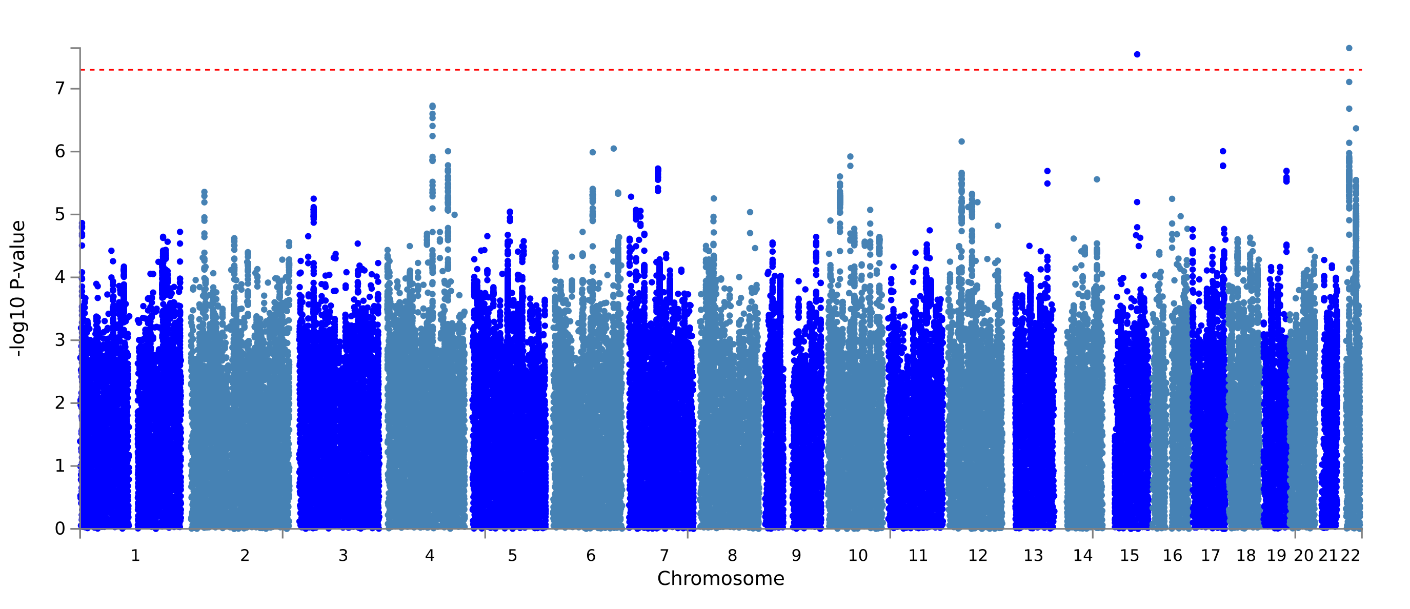


L. Milk tea intake


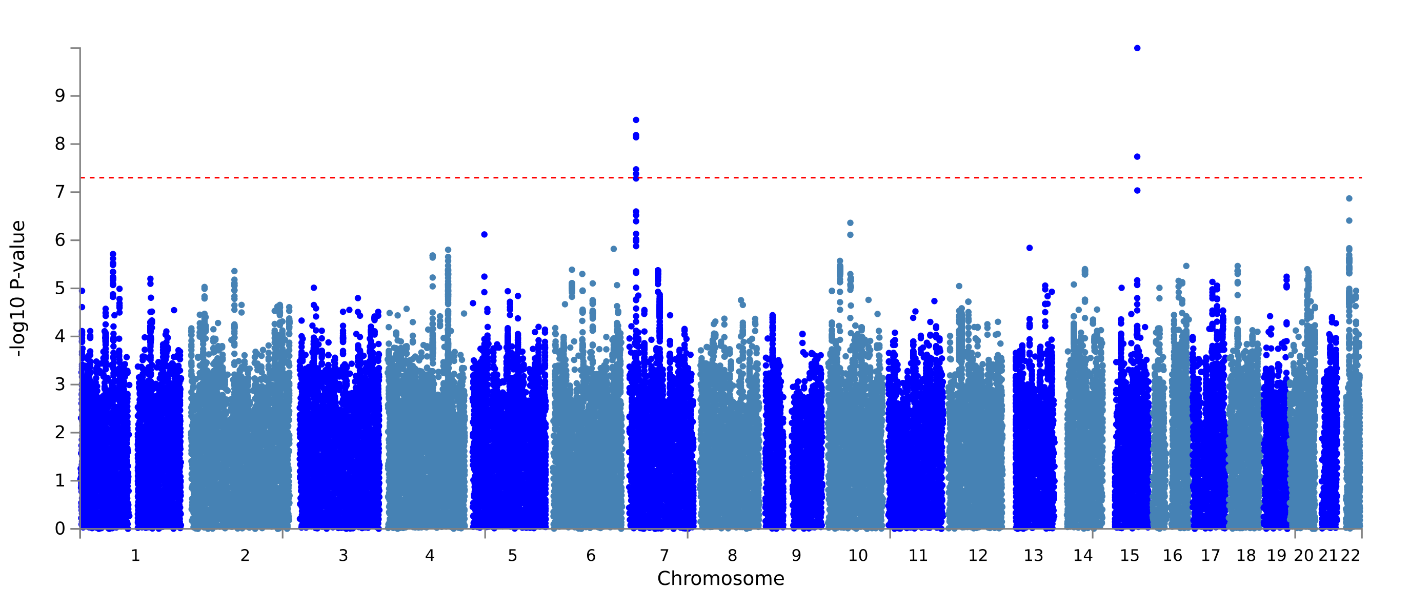


M. Caffeinated tea intake


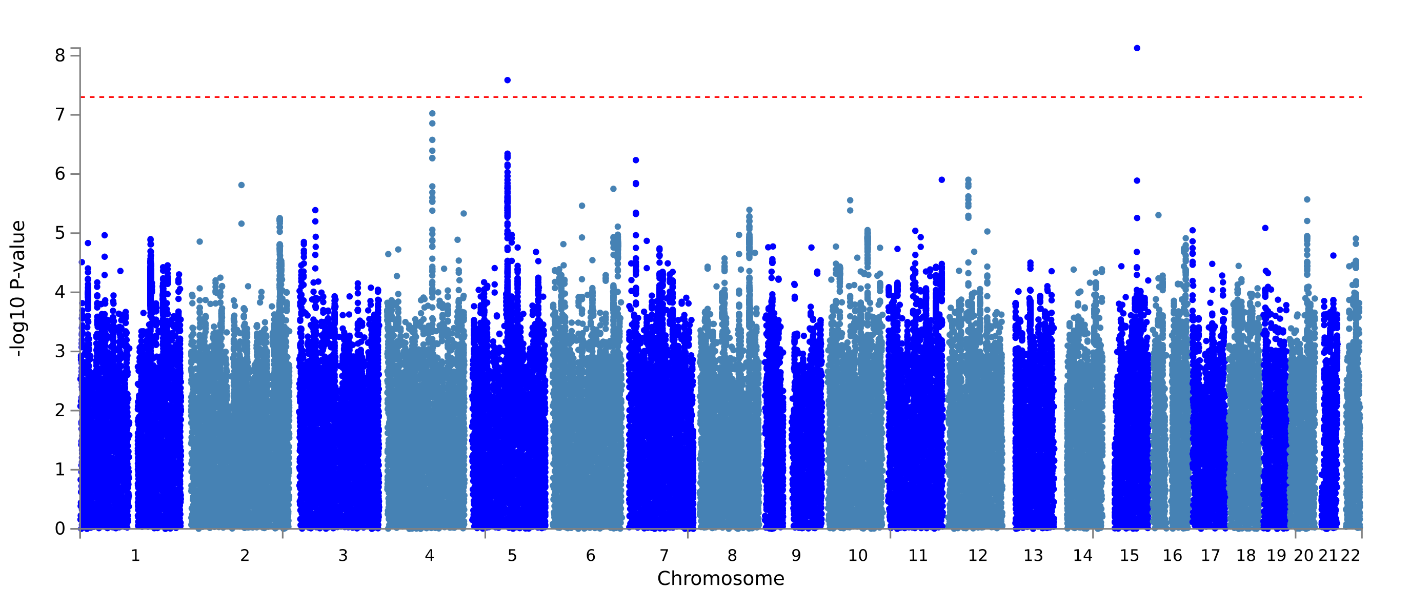


# Figure S2.

FUMA Regional plot of lead SNP rs3788372. Not shown are HRC reference panel SNPs, which FUMA is missing.


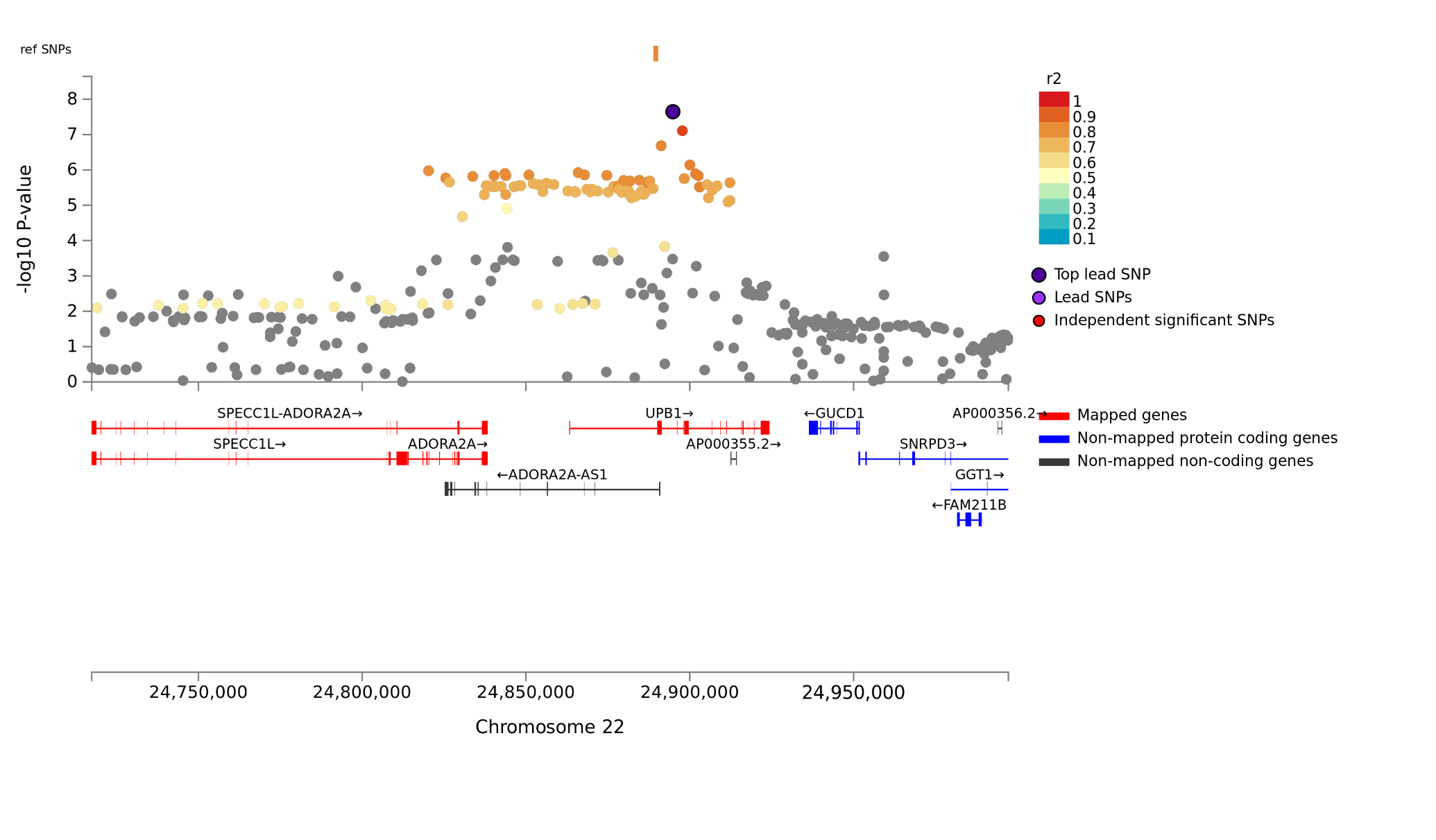


# Figure S3.

FUMA Regional plot of lead SNP rs2418224. Not shown are HRC reference panel SNPs, which FUMA is missing.


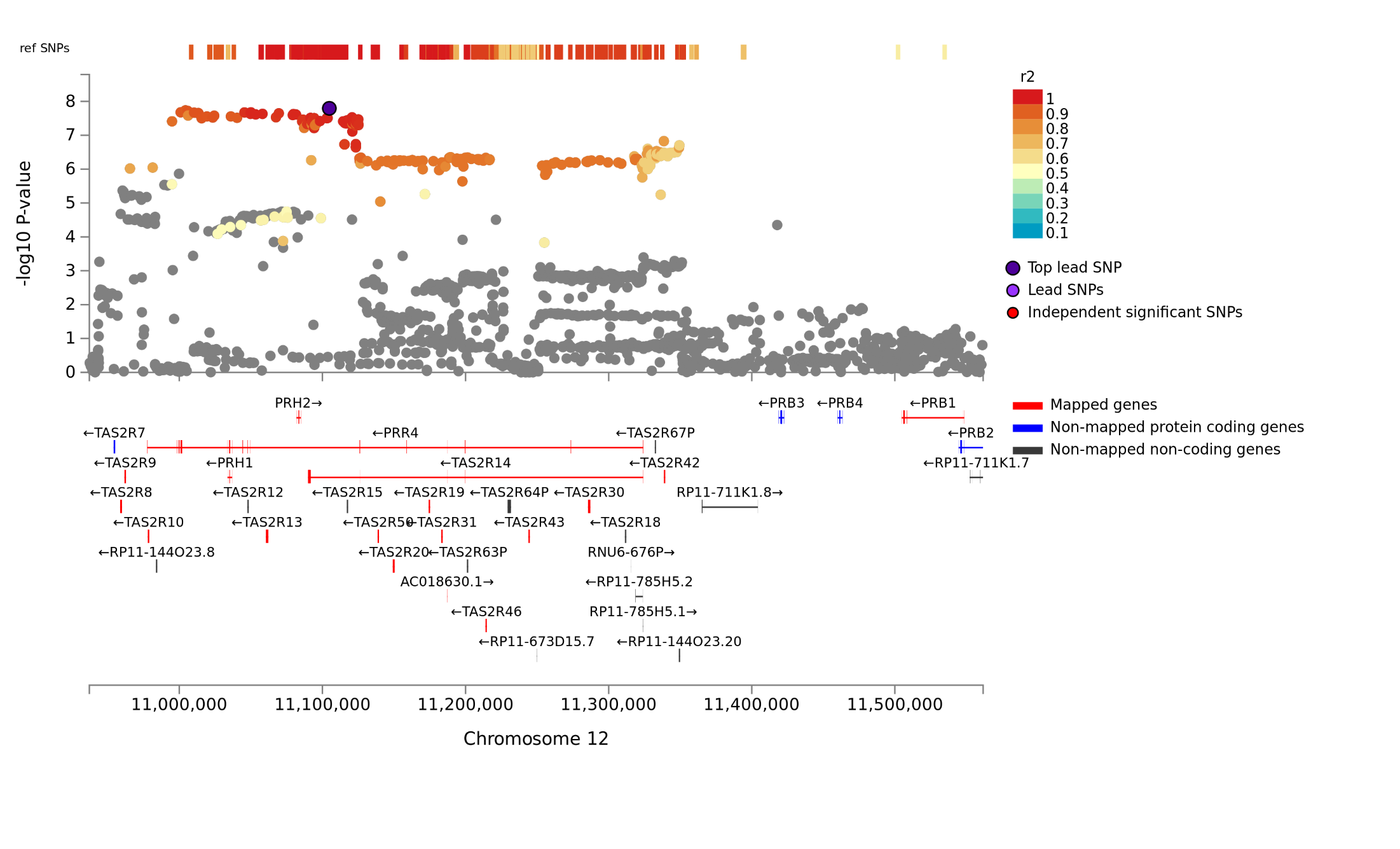

Supplement: Supplementary file 2 — Supplementary Information 2. [file 41598_2021_3153_MOESM2_ESM.docx]
